# Dementia risk factors and biomarkers in subjective cognitive decline: real world evidence from the monza brain health service

**DOI:** 10.1007/s10072-026-09426-1

**Published:** 2026-09-29

**Authors:** Federico Emanuele Pozzi, Francesca Solivani, Ilaria Vitagliano, Giovanni Isgrò, Giulia Remoli, Fatima Gabriela Macias Ortiz, Luca Cuffaro, Elisa Conti, Gessica Sala, Simona Andreoni, Chiara Paola Zoia, Valeria Cerina, Gianpaolo Basso, Lucio Tremolizzo, Ildebrando Appollonio, Carlo Ferrarese

**Affiliations:** 1 Neurology Department, Fondazione IRCCS San Gerardo dei Tintori, Via Pergolesi 33b, Monza, 20900 Italy; 2 Milan Center for Neuroscience (NeuroMI), University of Milano-Bicocca, Piazza dell’Ateneo Nuovo 1, Milan, 20126 Italy; 3 School of Medicine and Surgery, University of Milano-Bicocca, Via Cadore 48, Monza, 20900 Italy; 4 Tecnomed Foundation, Monza, 20900 Italy; 5 Neuroradiology Department, Fondazione IRCCS San Gerardo dei Tintori, Via Pergolesi 33b, Monza, 20900 Italy

**Keywords:** Alzheimer’s disease, Subjective Cognitive Decline, Blood based biomarkers, Prevention, Brain Health Services

## Abstract

**Aims:**

Up to 45% of dementia can be prevented by addressing modifiable risk factors. Brain Health Services (BHS) are emerging across Europe, targeting individuals with subjective cognitive decline (SCD) to assess, communicate and reduce their risk of dementia. Our aim is to characterize our BHS cohort, and assess differences in clinical, biological, neuroimaging, and impact of biomarker disclosure based on plasma Lumipulse p-Tau217.

**Methods:**

We analyzed baseline data from 186 consecutive individuals seen at our BHS (132 SCD). Cumulative dementia risk was calculated using the total Lancet relative risk (RR). Participants were grouped in plasma Lumipulse p-Tau217 above and below 0.15 pg/mL (A + and A–). Cluster analysis was performed with k-means.

**Results:**

The most prevalent potentially modifiable factors among participants with SCD were sleep impairment, smoking, physical inactivity, and hypertension. A + SCD subjects (32%) were older and showed worse performance on cognitive screening tests (*P*_*FDR*_ < 0.05). A + SCD showed higher GFAP and neurodegeneration on visual scales (*P*_*FDR*_ < 0.05). Two clusters were identified, one with low modifiable and biological risk and high cognition, and another with high risk and lower cognition. Disclosure was well accepted, with no differences between A– and A + in depression and anxiety.

**Discussion:**

Our findings showed a high prevalence of modifiable risk factors in SCD. A+ individuals demonstrated biological and structural hallmarks of early neurodegeneration. Biomarker disclosure was feasible, reflecting the potential benefits of transparent risk communication. These findings support incorporating plasma biomarkers and structured disclosure protocols in BHS, complementing multimodal prevention strategies to delay dementia onset.

**Supplementary Information:**

The online version contains supplementary material available at https://doi.org/10.1007/s10072-026-09426-1.

## Introduction

According to the latest work of the Lancet Commission on dementia prevention, intervention and care, up to 45% of dementia can be prevented by addressing 14 modifiable risk factors [1]. These factors have different impacts across the entire lifespan, and most of them are related to lifestyle.

Prevention is especially important for subjects at risk of developing cognitive impairment. The path towards dementia often starts with a preclinical phase of subjective cognitive decline (SCD), defined as a self-perceived decline in cognition despite normal performance on standard neuropsychological tests [2]. Depending on the setting, up to a third of individuals with SCD already harbor Alzheimer’s disease (AD), although SCD is a heterogeneous condition that may also be due to other neurodegenerative and non-neurodegenerative diseases [3]. Under the most recent Alzheimer’s Association (AA) Workgroup criteria, the diagnosis of AD may be established using plasma biomarkers, such as p-Tau217, provided that they show acceptable performance in the intended use population [4]. However, there is still debate on whether a diagnosis can be made in the SCD phase, and how to disclose it to patients. The 2024 AA Workgroup criteria define AD biologically, such that an individual with an abnormal Core 1 biomarker may meet the biological definition of AD irrespective of cognitive status. Nevertheless, the workgroup currently recommends against diagnostic testing in cognitively unimpaired individuals outside observational or therapeutic research studies. In contrast, the IWG retains a clinical-biological definition of AD and considers most biomarker-positive cognitively unimpaired individuals to be at risk for AD rather than affected by the disease, while reserving the term “presymptomatic AD” for uncommon profiles associated with a very high lifetime risk of progression. The IWG nevertheless permits biomarker investigation in subjective cognitive decliners within ad hoc non-diagnostic pathways aimed at estimating, communicating, and reducing future risk. Our BHS adopts such a non-diagnostic risk-communication pathway. Thus, biomarker testing occurs within the research context recognized by the AA Workgroup, whereas disclosure is aligned with the IWG risk-based approach [4, 5]. This approach aligns with current perspectives that emphasize interpreting blood-based biomarkers within a personalized risk-based framework rather than as standalone diagnostic tools [6].

SCD is a highly prevalent condition, an order of magnitude greater than MCI [7]. Yet, memory clinics are not designed to address the specific needs of these individuals, since they deal with diagnosis and treatment, rather than risk assessment and prevention. To address this issue, a new type of outpatient clinic has been proposed in recent years, the so-called Brain Health Services (BHS) [8]. These services are based on four pillars: risk of dementia assessment, risk communication, risk reduction, and cognitive enhancement. So far, more than 40 BHS have been founded across Europe, many of them in Italy. These BHS cover the whole spectrum from research projects to fully functioning clinical services [8]. The latter include our own BHS.

In the current study, our aim is to characterize our BHS cohort, and in particular SCD individuals, assessing clinical, risk factors, cognitive, biological, and neuroimaging differences based on plasma Lumipulse p-Tau217 above or below a pre-specified cut-off. We also aimed to evaluate the impact and feasibility of the combined disclosure protocol, including dementia risk, plasma biomarkers and APOE genotype.

## Methods

### Setting

A general presentation of our BHS is given elsewhere. In brief, subjects access the BHS through either self-referral (through our dedicated website or even word-of-mouth), primary care referral, or memory clinic referral, in the latter case after a diagnosis of SCD has been established. It is possible that some of these subjects have MCI at the time of the BHS visit, especially those accessing through self-referral, for which they are referred back to our memory clinic. Before the neurological visit, subjects complete online questionnaires on subjective cognitive decline – SCD Questionnaire (SCD-Q) [9], Everyday Cognition (ECOG) [10], mood (Hospital Anxiety and Depression Scale – HADS, Perceived Stress Scale – PSS), risk factors (including Ohio State University Traumatic Brain Injury Identification Method – OSU-TBI, Lubben Social Network Scale – LSNS, Pittsburgh Sleep Quality Index – PSQI, Epworth Sleepiness Scale – ESS, MEDI-LITE score [11], Geriatric Oral Health Assessment Index – GOHAI), cognitive reserve (Cognitive Reserve Index questionnaire – CRIq) [12] and resilience (Brief Resilience Scale – BRS). This is followed by an in-person evaluation with detailed history, cognitive screening with MoCA and quick MCI (qMCI), a brief screening of hand praxis (asking the subject to repeat four hand figures), and whisper test for hear loss [13]. Subjects are also asked to recall two recent facts heard in the news. A formal neuropsychological assessment was not yet available for all participants at the time of drafting this paper. Blood is then withdrawn for the assessment of blood biochemistry and blood-based biomarkers, including plasma Lumipulse p-Tau181, p-Tau217, NfL and GFAP, as well as APOE genotyping. Participants were categorized into plasma Lumipulse p-Tau217 above and below a cut-off of 0.15 pg/mL (A+ and A–, respectively), which demonstrated a 98% NPV and a 64% PPV for the detection of CSF-amyloid positivity in an independent SCD cohort from Barcelona [14]. Such groups do not equate to confirmed amyloid status. Subjects also undergo 3 Tesla MRI with assessment of visual scales, including mesial temporal atrophy (MTA), parietal cortical atrophy (PCA), global cortical atrophy (GCA), Fazekas scale [15], and cerebral microbleeds on SWI sequences.

A clinical diagnosis of SCD, MCI or functional cognitive decline (FCD) is based on established criteria [2, 16–18]. Please refer to the Supplementary Methods for further information. Subjects are considered worried-well (WW) if they are cognitively unimpaired individuals who are accessing the BHS because they are interested in prevention, rather than because of cognitive complaints. However, only if specifically asked, they may reveal a subjective impression of cognitive decline in specific domains, which they may attribute to aging and which they report as irrelevant in their everyday life.

Cumulative dementia risk is calculated using the total Lancet relative risk (Lancet RR), multiplying RRs for each factor from the 2024 Lancet Commission Report [1]. When reporting absolute and relative frequencies we considered the presence of risk factors irrespective of the age window in which they are thought to act. However, to avoid inflation of risk estimates, the specific factor RR is multiplied only if it is present during the relevant age window in which it is considered to influence dementia risk; for instance, air pollution RR contributes to the Lancet RR only in elderly subjects, as reported in the Lancet Commission Report [1]. To avoid underestimation of the risk, the Lancet RR is computed only when complete information on all the relevant factors is available. We acknowledge that the Lancet RR composite is not a validated epidemiological approach and that may lead to inflated estimates due to the potential non-independence of risk factors. However, this specific calculation was adopted following the framework for BHS, which emphasizes personalized risk communication over population-level statistics. In this clinical setting, the Lancet RR serves as a “risk multiplier” applied to a baseline risk [19]. We also calculated other validated scores for the appropriate age categories [20], including CAIDE (39–64 years) [21], BDSI (> 64 years) [22] and LIBRA (> 55 years) [23], as well as the sum of the risk factors in the Lancet Commission Report [1].

Then, subjects are called again for a second visit, online or in-person depending on their preference, in which the results of biomarkers (only p-Tau217 and APOE genotype) and the risk profile are disclosed, and tailored recommendations on risk reduction are proposed. The actual risk communicated to the subject is obtained by multiplying the Lancet RR for the baseline risk calculated with Brookmeyer’s method [19, 24], using Italian mortality data instead of United States ones. A week after disclosure, subjects complete online questionnaires, including HADS and the Impact of Event Scale – Revised (IES-R). The complete protocol for disclosure and risk reduction is presented in the Supplementary Methods.

Our BHS utilizes a dual-purpose operational model, integrating a clinical prevention service providing risk assessment, communication and reduction to the individuals, while also coordinating a large network of Lombardy BHS with harmonized procedures for risk assessment and communication, united under a multicenter prospective observational cohort study aiming to characterize SCD. Nevertheless, individual centers within this network retain clinical autonomy in defining and implementing specific risk-reduction strategies, ensuring that interventions remain personalized and responsive to the patient’s unique profile and local clinical resources.

For the current work, we analyzed all available baseline cross-sectional data from all consecutive individuals seen at our BHS up to March 16th, 2026, with a focus on SCD subjects. Data on other groups (FCD, MCI and WW) are presented to provide a comprehensive, real-world overview of the total heterogeneous BHS population, rather than to suggest that the study was powered to detect differences between these specific subsets. These groups should therefore be regarded as descriptive “incidental” clinical populations that enter the BHS.

### Plasma biomarkers and APOE genotype

Blood samples (5 ml) were obtained from all patients after over-night fasting in EDTA K2-coated tubes and immediately centrifuged (2000 g, 10 min, RT). Plasma aliquots were frozen at − 80 °C until blind assessments. Plasma p-Tau181, p-Tau217, GFAP and NfL were measured by using commercially available kits with the Lumipulse^®^G600II platform (Fujirebio). To analyze APOE genotype, total DNA was extracted from peripheral blood using a commercial DNA extraction kit (Qiagen, Venlo, Netherlands), and the genotype was determined using DiaPlexQTM Apolipoprotein E (ApoE) Genotyping Kit (SolGent, Daejeon, Korea).

### MRI protocol

MRIs were acquired on a 3.0 T Philips Ingenia CX (Philips Medical Systems Nederland B.V. of Eindhoven) equipped with a 32-channel head coil, at the Neuroradiology Department of Fondazione IRCCS San Gerardo dei Tintori. MR data acquisition lasted 30 min. Whole brain structural MR images were acquired using volumetric magnetization prepared rapid gradient-echo sequence 3D T2 1 mm thickness isotropic FLAIR, 3D SWI, T2 TSE and axial EPI DWI, 3D pseudo-continuous arterial spin labeling, with 1 mm × 1 mm in plane resolution and 1 mm slice thickness. The protocol also included 3D T2 1 mm thickness isotropic FLAIR, 3D susceptibility-weighted imaging, T2 TSE, axial echo-planar imaging and DWI, as well as 3D pCASL sequence with a single-shot echo-planar imaging readout.

### Statistical analysis

Results are presented as frequencies (percentages) or median and interquartile range. Differences among groups were tested with χ²-square test for categorial variables, and Kruskal-Wallis rank sum test or Wilcoxon rank sum test for continuous variables. For plasma biomarker trends across different diagnostic groups, we used a post-hoc analysis with the Jonckheere–Terpstra test, with the order FCD < WW < SCD < MCI. Differences in cognitive variables were adjusted for age, sex and education, while differences in risk factors, biomarkers and MRI scales were adjusted for age. For the clustering analysis of the SCD subjects, we selected the following variables: age, plasma biomarkers (p-Tau217, GFAP, NfL), Lancet RR, and qMCI. All variables were standardized prior to clustering. K-means clustering was performed to identify subgroups of participants based on biomarker and clinical profiles. The optimal number of clusters was determined using the silhouette method, and cluster stability was evaluated using the clusterboot procedure with 1000 bootstrap samples. Principal Component Analysis (PCA) was used to visualize cluster separation in two dimensions, and variable contributions to the principal components were assessed. Differences between clusters for each variable were assessed using Wilcoxon rank-sum tests, and effect sizes have been computed with rank-biserial correlation (r).

An exploratory linear regression modeled IES-R total as a function of A status, age, and APOE ε4 carriage.

All *P-*values were corrected for false discovery rate (FDR) to account for multiple comparisons.

## Results

### Whole sample

The whole cohort seen comprised 186 patients. Of these, 132 were SCD, 19 MCI, 18 had FCD and 17 were WW. Supplementary Fig. S1 shows the number of subjects in each category in the whole cohort and in the subset with plasma biomarkers available.

A number of significant differences emerged across diagnostic groups, in particular in sex, age, education, cognitive complaints duration, perception of own memory compared to peers, complaints domains, Lancet RR, mood impairment, depression, perceived stress, social isolation, sleep impairment, cognitive measures (all *P*_*FDR*_ < 0.05 for the among-groups difference), shown in Table 1 and Supplementary Table S1. FCD were almost always females, they were younger (53.00 (49.25, 57.00) years), reported the longest duration of cognitive impairment (4.00 (2.00, 5.75) years), and considered their memory worse than peers in 89% of the cases, which was the highest percentage across diagnostic groups. They also reported more frequently issues in domains beyond memory, such as attention (83%) and language (94%). FCD subjects showed more frequently mood impairment (83%) and depression (28%), and had the highest values of anxiety and depression on HADS (12.00 (11.00, 16.00)), as well as the highest perceived stress on the PSS (22.00 (19.75, 24.50)). FCD individuals more frequently reported social isolation, both currently (72%) and in the past (82%). Finally, they complained more frequently about sleep impairment (78%), which resulted in the highest scores on the PSQI and the ESS, although no significant among-groups differences emerged on these scales.

**Table 1:** Characteristics of the whole cohort

Variable	Whole cohort		WW		FCD		SCD		MCI				
	*N*	Distribution	*n*	Distribution	*n*	Distribution	*n*	Distribution	*n*	Distribution	*P*	*P*_adj_	*P*_FDR_
*Clinical variables*													
Sex [Female]	186	113 (60.75%)	17	7 (41.18%)	18	17 (94.44%)	132	79 (59.85%)	19	10 (52.63%)	0.008		0.025
Age	186	67.00 (60.00, 72.00)	17	64.00 (59.00, 66.00)	18	53.00 (49.25, 57.00)	132	68.00 (61.00, 72.00)	19	77.00 (71.50, 79.50)	< 0.001		< 0.001
Education [years]	186	13.00 (11.00, 16.00)	17	13.00 (13.00, 16.00)	18	13.00 (12.25, 17.50)	132	13.00 (11.00, 16.00)	19	11.00 (8.00, 12.50)	0.003		0.014
Complaints duration [years]	185	2.00 (1.50, 4.00)	17	0.00 (0.00, 0.00)	18	4.00 (2.00, 5.75)	131	2.00 (2.00, 4.00)	19	2.00 (1.88, 5.00)	< 0.001		< 0.001
*Complaints*													
Memory	186	178 (95.70%)	17	13 (76.47%)	18	18 (100.00%)	132	128 (96.97%)	19	19 (100.00%)	< 0.001		0.004
Attention	178	102 (57.30%)	17	2 (11.76%)	18	15 (83.33%)	125	75 (60.00%)	18	10 (55.56%)	< 0.001		0.002
Orientation	186	49 (26.34%)	17	1 (5.88%)	18	8 (44.44%)	132	35 (26.52%)	19	5 (26.32%)	0.082		0.170
Language	186	124 (66.67%)	17	7 (41.18%)	18	17 (94.44%)	132	89 (67.42%)	19	11 (57.89%)	0.008		0.025
*Risk factors*													
Depression	186	19 (10.22%)	17	1 (5.88%)	18	5 (27.78%)	132	9 (6.82%)	19	4 (21.05%)	0.015		0.041
Social isolation	185	65 (35.14%)	17	4 (23.53%)	18	13 (72.22%)	131	42 (32.06%)	19	6 (31.58%)	0.006		0.021
Lancet Commission RR	131	3.71 (2.43, 5.67)	15	2.91 (1.94, 3.81)	12	4.27 (3.71, 6.67)	92	3.63 (2.19, 5.46)	12	6.62 (3.73, 9.26)	0.003		0.015
*Cognitive variables*													
MyCog	129	11.00 (8.00, 15.00)	14	6.50 (0.50, 9.75)	12	15.00 (12.25, 16.50)	87	11.00 (8.00, 13.50)	16	14.00 (11.50, 16.00)	< 0.001	< 0.001	0.005
TheirCog	69	10.00 (5.00, 14.00)	5	0.00 (0.00, 5.00)	7	13.00 (6.50, 15.50)	42	9.00 (6.00, 12.75)	15	15.00 (9.50, 19.00)	0.011	0.015	0.032
MoCA	181	25.00 (24.00, 28.00)	16	26.50 (25.00, 28.00)	18	26.00 (24.25, 28.75)	128	26.00 (24.00, 28.00)	19	20.00 (18.00, 21.50)	< 0.001	< 0.001	< 0.001
qMCI	142	61.75 (51.62, 72.38)	16	71.00 (62.75, 74.25)	12	67.00 (57.62, 81.00)	99	62.00 (53.50, 71.75)	15	41.00 (39.25, 43.50)	< 0.001	< 0.001	< 0.001
*Plasma biomarkers*													
pTau-217	115	0.11 (0.08, 0.15)	12	0.09 (0.08, 0.11)	6	0.07 (0.06, 0.08)	84	0.11 (0.08, 0.15)	13	0.18 (0.11, 0.47)	0.002		0.008
pTau-181	115	0.87 (0.69, 1.29)	12	0.82 (0.70, 0.99)	6	0.80 (0.74, 0.88)	84	0.85 (0.69, 1.29)	13	1.07 (0.94, 1.88)	0.118		0.228
GFAP	115	42.00 (30.20, 64.00)	12	46.50 (28.43, 50.75)	6	32.70 (29.30, 56.57)	84	38.30 (29.50, 58.50)	13	74.90 (49.00, 96.40)	0.022		0.053
NfL	115	15.96 (11.54, 20.52)	12	16.00 (12.66, 18.87)	6	10.44 (7.50, 13.07)	84	15.63 (11.45, 20.00)	13	22.17 (20.28, 24.79)	< 0.001		0.004

Characteristics of the whole cohort. Data are expressed as median with interquartile range or percentage (%). Differences among diagnostic groups were tested with the Kruskal-Wallis rank sum test or a Pearson’s *χ²* test, depending on the type of variable. Cognitive variables were compared adjusting for age, sex, and education (*P*_*adj*_)

*P-*values were adjusted for multiple comparisons with FDR (*P*_*FDR*_*)*

Due to limited sample sizes, comparisons involving FCD, MCI and WW groups are descriptive and hypothesis-generating only. Statistical values for these rows should be interpreted with caution

As expected, WW reported the shortest duration of cognitive complaints, and none of them considered their memory worse than that of their peers, although when probed they admitted to have memory issues in 76% of the cases. WW also showed the lowest scores on self-rated and informant-rated cognition (MyCog 6.50 (0.50, 9.75); TheirCog 0.00 (0.00, 5.00)), and the least prevalent physical inactivity (18%).

MCI subjects were the oldest (77.00 (71.50, 79.50) years) and least educated group (11.00 (8.00, 12.50) years). They were also the group with the highest Lancet RR (6.62 (3.73, 9.26)) and the most prevalent physical inactivity (74%). They also scored the worst on all cognitive screening measures, including MoCA (20.00 (18.00, 21.50)), qMCI (41.00 (39.25, 43.50)), clock drawing test (CDT, impaired in 58% of cases), and recall of general recent facts (1.50 (1.00, 2.00)).

Air-pollution exposure was assigned to all participants on the basis of the geographic setting and therefore did not discriminate between study groups.

Plasma biomarkers were available in a subset of 115 individuals. Significant among-group differences emerged on plasma Lumipulse p-Tau217 and NfL (all *P*_*FDR*_ < 0.05 for the among-groups difference). A gradient was observed, with levels of biomarker being lowest among FCD, intermediate in SCD and WW, and highest in MCI. The Jonckheere–Terpstra test confirmed this trend for all biomarkers (plasma Lumipulse p-Tau217 *P*_*FDR*_ < 0.001, p-Tau181 *P*_*FDR*_ = 0.038, GFAP *P*_*FDR*_ = 0.019, NfL *P*_*FDR*_ < 0.001).

### SCD subgroup

**Fig. 1:**
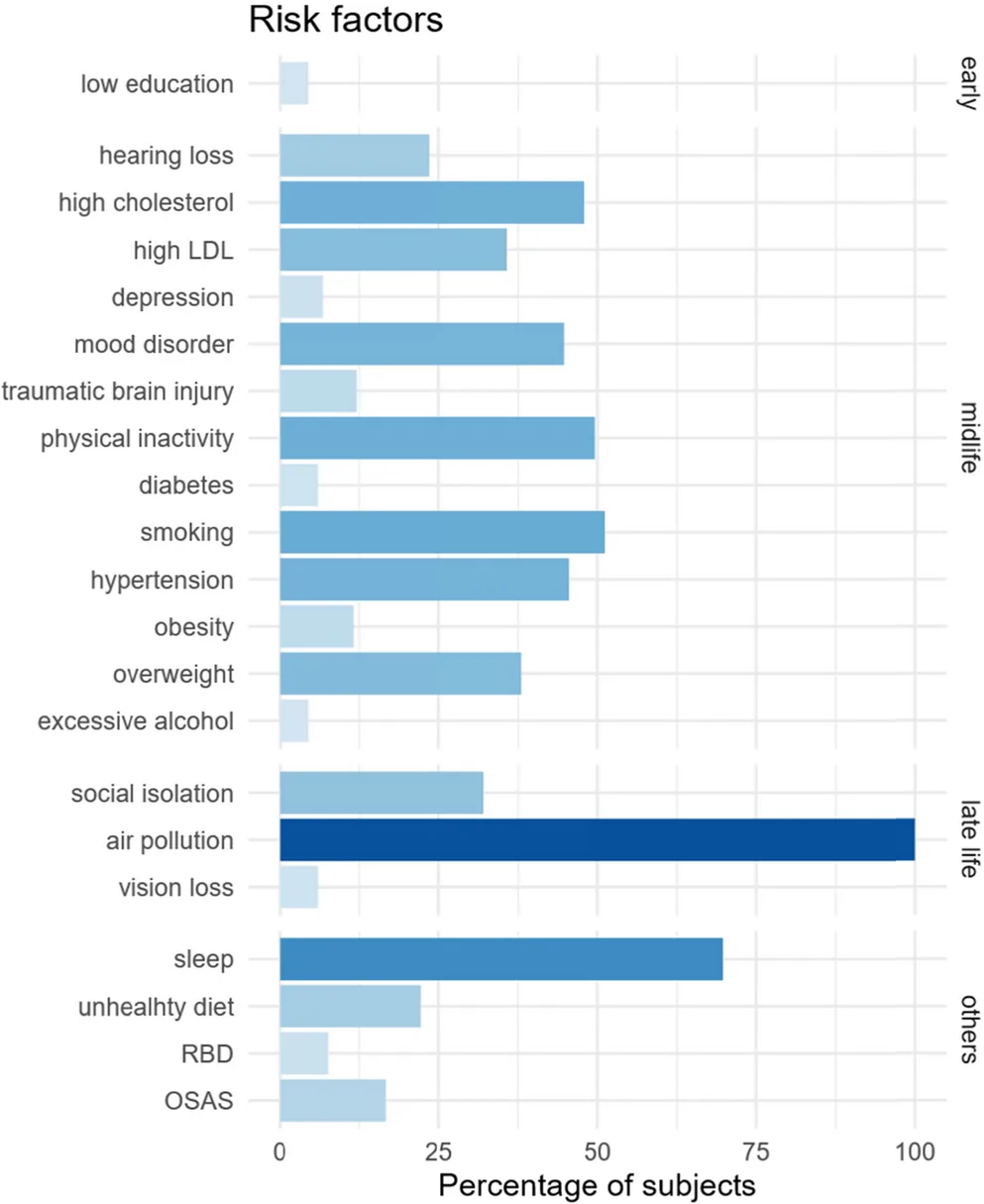
Prevalence of risk factors for dementia in the SCD subgroup. Factors are grouped based on the life epoch subdivision reported in the 2024 Lancet Commission. Other factors not included in the Lancet Commission include high cholesterol, being overweight, mood disorder, sleep impairment, RBD, OSAS and unhealthy diet. Abbreviations: *OSAS* – Obstructive sleep apnea syndrome; *RBD* – REM-behavior sleep disorder

For subsequent analyses, we focused on the SCD subgroup (*n* = 132). The prevalence of risk factors among SCD is shown in Fig. 1. Among participants with SCD, the most prevalent potentially modifiable factors were smoking (51%), physical inactivity (50%), and hypertension (46%). Among putative risk factors, sleep impairment, defined as either self-reported sleep issues, suspected or confirmed RBD, or OSAS, was the most prevalent (70%).

The Lancet RR showed a strong positive correlation with the cumulative number of modifiable dementia risk factors (*n* = 92, Spearman *ρ* = 0.54, *P* < 0.001), supporting preservation of participant ranking despite the weighted nature of the composite score, as well as with the LIBRA score (*n* = 81, Spearman *ρ* = 0.64, *P* < 0.001) and BDSI (*n* = 55, Spearman *ρ* = 0.31, *P* = 0.020). A trend for correlation with CAIDE (*n* = 37, Spearman *ρ* = 0.30, *P* = 0.069) was also noted.

Among SCD individuals with available plasma biomarkers (*n* = 84), 27 (32%) were A+ according to plasma Lumipulse p-Tau217. As shown in Table 2 and Supplementary Table S2, A+ individuals were older (72.00 (68.00, 76.00) vs. 64.00 (60.00, 69.00), *P*_*FDR*_ < 0.001). There were no differences in complaints across cognitive domains.

**Table 2:** Differences between A + and A- subjects

Variable	Whole SCD cohort		A-		A+					
	*n*	distribution	*n*	distribution	*n*	distribution	*P*	*P*_adj_	*P*_FDR_	*r*
*Clinical variables*										
Sex (F)	84	52 (61.90%)	57	39 (68.42%)	27	13 (48.15%)	0.122		0.385	
Age	84	67.50 (60.75, 71.00)	57	64.00 (60.00, 69.00)	27	72.00 (68.00, 76.00)	< 0.001		< 0.001	0.58
Education (years)	84	14.00 (13.00, 17.00)	57	14.00 (13.00, 17.00)	27	13.00 (13.00, 17.00)	0.516		0.852	-0.09
*Risk factors*										
Whisper test	81	5.00 (3.50, 5.50)	55	5.00 (4.25, 5.50)	26	3.75 (2.62, 4.88)	0.001	0.178	0.012	-0.44
Lancet Commission RR	82	3.71 (2.30, 5.46)	55	3.22 (1.94, 4.98)	27	5.22 (2.97, 6.48)	0.012	0.882	0.074	0.34
*Cognitive variables*										
MyCog	76	11.00 (8.00, 13.00)	49	10.00 (8.00, 12.00)	27	12.00 (8.50, 14.50)	0.199	0.351	0.498	0.18
TheirCog	37	9.00 (6.00, 13.00)	21	9.00 (6.00, 13.00)	16	10.00 (4.00, 13.25)	1.000	0.837	1.000	0.00
MoCA	83	26.00 (25.00, 28.00)	56	26.00 (25.00, 28.00)	27	26.00 (23.50, 28.00)	0.128	0.069	0.389	-0.21
qMCI	81	64.00 (55.00, 72.00)	55	67.00 (60.00, 74.75)	26	59.50 (48.75, 63.75)	< 0.001	< 0.001	0.004	-0.49
*Plasma biomarkers*										
pTau-217	84	0.11 (0.08, 0.15)	57	0.09 (0.08, 0.11)	27	0.23 (0.16, 0.48)	< 0.001	< 0.001	< 0.001	1.00
pTau-181	84	0.85 (0.69, 1.29)	57	0.78 (0.65, 0.88)	27	1.32 (1.00, 1.83)	< 0.001	< 0.001	< 0.001	0.74
GFAP	84	38.30 (29.50, 58.50)	57	34.40 (27.30, 43.90)	27	64.20 (43.65, 93.75)	< 0.001	< 0.001	< 0.001	0.66
NfL	84	15.63 (11.45, 20.00)	57	14.68 (10.96, 17.82)	27	18.16 (13.98, 25.12)	0.009	0.401	0.061	0.35
APOE4 carrier	84	27 (32.14%)	57	17 (29.82%)	27	10 (37.04%)	0.681		0.962	0.07
*MRI – visual scales*										
MTA [mean]	46	0.50 (0.00, 1.00)	28	0.00 (0.00, 0.50)	18	1.00 (0.25, 1.50)	0.003	0.187	0.026	0.49
GCA	46	0.00 (0.00, 1.00)	28	0.00 (0.00, 0.25)	18	1.00 (1.00, 2.00)	< 0.001	0.010	< 0.001	0.65
PCA [mean]	46	1.00 (0.00, 1.00)	28	0.00 (0.00, 1.00)	18	1.00 (1.00, 1.88)	< 0.001	0.010	< 0.001	0.65
Fazekas	45	1.00 (1.00, 2.00)	28	1.00 (0.00, 1.00)	17	1.00 (1.00, 2.00)	0.042	0.530	0.192	0.34
Microbleeds	45	0.00 (0.00, 0.00)	28	0.00 (0.00, 0.00)	17	0.00 (0.00, 0.00)	0.463	0.581	0.812	-0.04

Characteristics of the SCD cohort. Data are expressed as median with interquartile range or percentage (%). A+ was defined as plasma p-Tau217 ≥ 0.15 pg/mL, measured by Lumipulse (Fujirebio). Differences between A+ and A– groups were tested with the Wilcoxon rank sum test or a Pearson’s *χ²* test, depending on the type of variable. Risk factors, plasma biomarkers and MRI visual scales *P-*values were compared adjusting for age (*P*_*adj*_). Cognitive variables were compared adjusting for age, sex, and education (*P*_*adj*_). *P-*values were adjusted for multiple comparisons with FDR. Effect sizes have been computed with rank-biserial correlations (r)

Abbreviations: *GCA*, Global Cortical Atrophy; *MTA*, Medial Temporal Atrophy; *PCA*, Parietal Cortical Atrophy

There were no differences in specific risk factors, except for hearing loss screening with the whisper test (worse in A+, *P*_*FDR*_ = 0.012; the difference was no longer significant after adjusting for age). The Lancet RR was higher in A+ subjects (5.22 (2.97, 6.48) vs. 3.22 (1.94, 4.98)), but the difference was no longer significant after adjusting for age, nor after FDR correction.

A+ subjects performed worse on the qMCI, albeit within normal range (59.50 (48.75, 63.75) vs. 67.00 (60.00, 74.75), *P*_*FDR*_ = 0.004). As expected, A+ had higher levels of plasma Lumipulse p-Tau217 and p-Tau181, but also of plasma Lumipulse GFAP (all *P*_*FDR*_ < 0.001). The difference in plasma NfL levels was marginally significant after FDR correction (*P*_*FDR*_ = 0.061) and not significant after adjusting for age. A+ subjects also showed higher neurodegenerative changes on MRI visual scales, including MTA, GCA and PCA (all *P*_*FDR*_ <0.05). Only the differences in GCA and PCA were significant after adjusting for age.

**Fig. 2:**
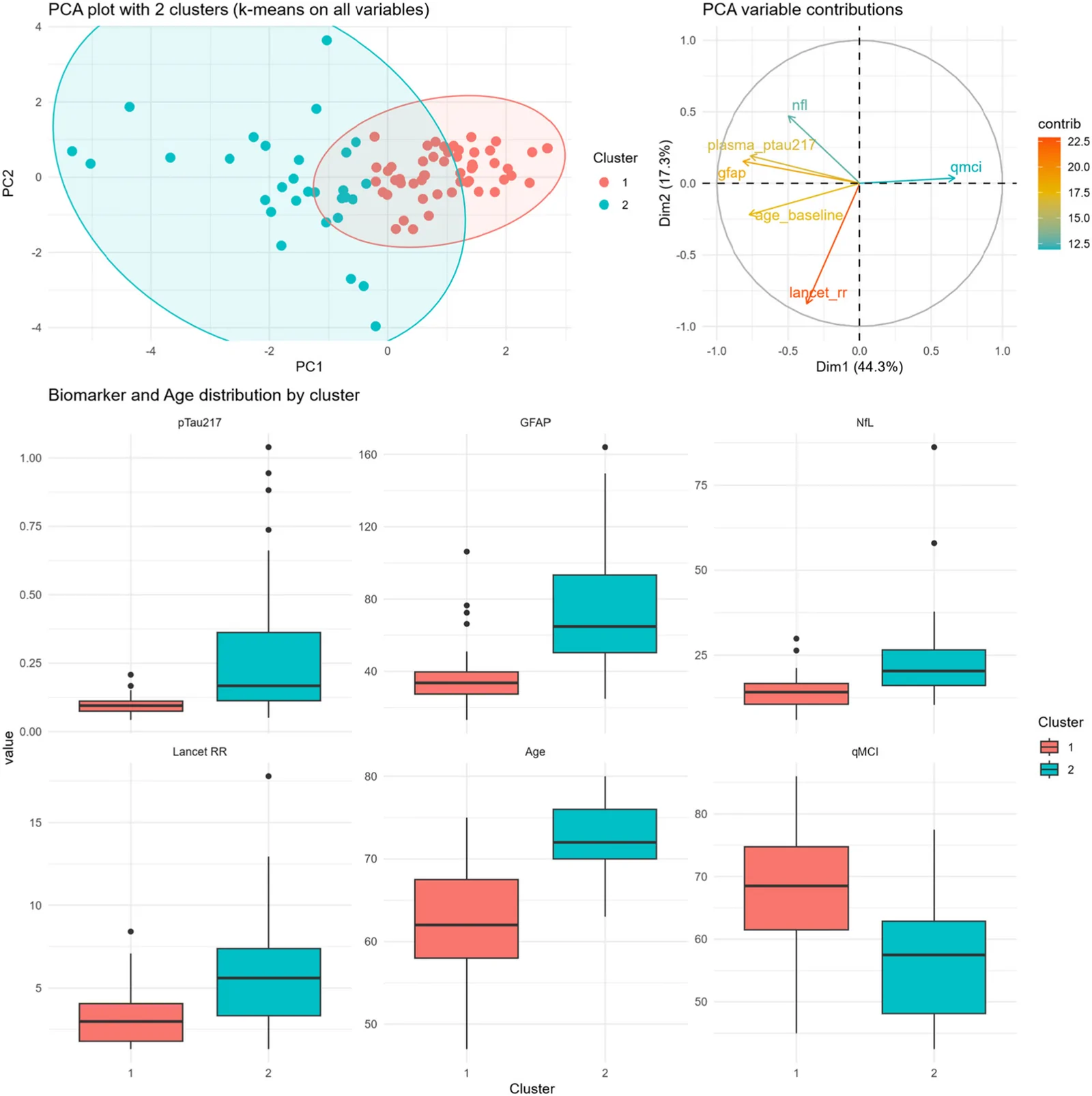
Combined plot showing principal component analysis with 2 clusters (top left), and variable contributions (top right), and boxplots of variable distributions by clusters (bottom)

Given the relatively small sample size (*n* = 84), we selected only 6 variables for clustering analysis, trying to capture the heterogeneous dimensions of SCD: plasma Lumipulse p-Tau217, GFAP and NfL (reflecting amyloid-β pathology, neuroinflammation and neurodegeneration, respectively), Lancet RR as a measure of modifiable risk, age and qMCI to reflect cognition. The silhouette method identified 2 clusters (see Supplementary Fig. S2), shown in Fig. 2.

Cluster 1 (*n* = 51) included younger participants with lower biomarker values, lower Lancet RR and higher qMCI scores. Cluster 2 (*n* = 30) included older participants with higher biomarker levels, higher Lancet RR and lower qMCI scores (see also Supplementary Table S3). The PCA plot demonstrated clear separation along PC1, reflecting differences in age and neurodegeneration biomarkers. The radar chart (Supplementary Figure S3) showed that Cluster 2 had higher standardized values for all variables except qMCI, which was lower. Internal validation showed a mean silhouette width of 0.452, indicating a moderate but meaningful cluster structure. Bootstrap-based cluster stability analysis yielded mean Jaccard similarity coefficients of 0.625 for Cluster 1 and 0.824 for Cluster 2, indicating moderate stability of the low-risk cluster and good stability of the high-risk cluster. A sensitivity analysis clustering based on GFAP, NfL, Lancet RR, age, and qMCI, excluding p-Tau217, yielded nearly identical assignments (only 12 participants of Cluster 1 were reassigned to Cluster 2), indicating that cluster structure was robust and not solely driven by p-Tau217.

We further compared A− subjects from the original clusters (see Supplementary Table S4). Among A− individuals, 10 (18%) were assigned to the original high-risk cluster (Cluster 2). Compared with A− individuals in Cluster 1, A−/Cluster 2 participants were older, showed lower qMCI scores, higher Lancet RR, and increased plasma Lumipulse GFAP and NfL (all *P <* 0.05). Hypertension was also more prevalent in Cluster 2 (*P* = 0.006). However, after correction for multiple comparisons, the only significant difference was age (*P*_*FDR*_ < 0.001). Plasma Lumipulse p-Tau217 levels were comparable between groups (*P* = 0.377), supporting the absence of amyloid-associated biomarker elevation despite increased risk and neuroinflammatory signatures.

### Biomarker disclosure

Biomarker results were disclosed to 42 subjects with SCD so far, of which 10 (24%) were A+. The results of the disclosures are shown in Table 3 and Supplementary Table S5. Almost all subjects considered the disclosure to be useful and informative, and they were generally satisfied with how it was conducted (satisfaction was rated on 5-point Likert scale). Only one subject expressed the need for psychological support. Around two thirds of the subjects were more motivated to change their lifestyle after disclosure, with no differences between A+ and A−. Risk of dementia perception, rated on a 5-point Likert scale, decreased in A– subjects and did not change in A+ after disclosure; the difference was not significant after FDR correction.

**Table 3:** Main disclosure-related differences between A + and A- SCD subjects

Variable	Whole SCD cohort		A-		A+			
	*n*	distribution	*n*	distribution	*n*	Distribution	*P*	*P*_FDR_
Mode [in person]	42	25 (59.52%)	32	17 (53.12%)	10	8 (80.00%)	0.253	0.453
Satisfaction	42	5.00 (4.00, 5.00)	32	5.00 (4.75, 5.00)	10	5.00 (4.00, 5.00)	0.238	0.453
Enough information	42	41 (97.62%)	32	31 (96.88%)	10	10 (100.00%)	1.000	1.000
Risk perception pre-disclosure	42	3.00 (2.00, 3.00)	32	3.00 (2.00, 4.00)	10	3.00 (2.25, 3.00)	0.258	0.453
Risk perception post-disclosure	42	2.00 (2.00, 3.00)	32	2.00 (2.00, 3.00)	10	3.00 (2.25, 3.00)	0.016	0.056
Risk perception difference	42	0.00 (-1.00, 0.00)	32	-1.00 (-2.00, 0.00)	10	0.00 (0.00, 0.00)	0.013	0.056
Need for psychological support	42	1 (2.38%)	32	1 (3.12%)	10	0 (0.00%)	1.000	1.000
Lifestyle change motivation	42	25 (59.52%)	32	18 (56.25%)	10	7 (70.00%)	0.455	0.585
IES-R – avoidance	42	0.44 (0.13, 0.75)	32	0.32 (0.00, 0.63)	10	0.88 (0.53, 1.16)	0.010	0.056
IES-R – intrusions	42	0.25 (0.00, 0.72)	32	0.25 (0.00, 0.53)	10	0.69 (0.50, 1.16)	0.011	0.056
IES-R – hyperarousal	42	0.00 (0.00, 0.33)	32	0.00 (0.00, 0.21)	10	0.08 (0.00, 0.70)	0.285	0.453
IES-R – sum of means	42	0.79 (0.28, 1.49)	32	0.62 (0.10, 1.16)	10	1.66 (1.09, 2.81)	0.012	0.056
IES-R – total	42	6.00 (2.00, 11.00)	32	4.50 (0.75, 10.00)	10	11.50 (7.25, 21.25)	0.013	0.056
HADS – anxiety, difference	38	-1.00 (-2.00, 0.00)	30	-1.00 (-2.00, 0.00)	8	-0.50 (-1.25, 2.25)	0.281	0.453
HADS – depression, difference	38	-1.00 (-3.00, 1.00)	30	-1.50 (-3.00, 1.00)	8	0.00 (-1.25, 1.25)	0.278	0.453
HADS – total, difference	38	-1.00 (-4.00, 0.75)	30	-2.00 (-4.75, 0.00)	8	1.00 (-1.00, 2.00)	0.075	0.202

Differences in disclosure variables between A+ and A– SCD. Data are expressed as median with interquartile range or percentage (%). A+ was defined as plasma p-Tau217 ≥ 0.15 pg/mL, measured by Lumipulse (Fujirebio). Differences between A+ and A– groups were tested with the Wilcoxon rank sum test or a Pearson’s χ² test, depending on the type of variable. *P*-values were adjusted for multiple comparisons with FDR

Abbreviations: *HADS*, Hospital Anxiety and Depression Scale; *IES-R*, Impact of event scale – revised

Although total HADS was generally lower in A− and higher in A+ compared to pre-disclosure, there were no significant differences in the variation of HADS score and subscores after disclosure. A+ subjects showed higher scores on the IES-R sum of means (1.66 (1.09, 2.81) vs. 0.62 (0.10, 1.16)), driven by avoidance and intrusions subscores, and IES-R total (11.50 (7.25, 21.25) vs. 4.50 (0.75, 10.00)). However, such differences were only marginally significant after FDR correction (all *P*_*FDR*_ = 0.056).

Among the 42 participants who completed the disclosure assessment, APOE ε4 was present in 4/10 (40.0%) A+ and 10/32 (31.3%) A− (Fisher’s exact test *P* = 0.707). In an exploratory multivariable sensitivity analysis adjusting for age and APOE ε4 carriage (adjusted R^2^ = 0.29, *P* < 0.001, intercept = -43.93, SE = 15.8), A status was not associated with IES-R total (unstandardized *β* = 1.13, SE = 4.10, *P* = 0.785). Higher age (unstandardized *β* = 0.75, SE = 0.23, *P* = 0.003) and APOE ε4 carriage (unstandardized *β* = 8.87, SE = 3.30, *P* = 0.011) were associated with higher IES-R scores. The association between APOE and IES-R total persisted also when age was removed from the model.

## Discussion

We presented one of the largest BHS cohorts in Europe so far, comprising 186 individuals accessing our BHS. The most common diagnosis was SCD, followed by MCI, FCD and WW. Our findings can be summarized as follows. First, our results show peculiar cognitive and clinical profiles for each group, albeit differences should be regarded as exploratory in nature due to the limited sample size. In particular, FCD are typically younger, more frequently females, with depressive symptoms and social isolation. MCI are on average older, with higher biomarkers of amyloid, neurodegeneration and neuroinflammation, and worse cognition. WW, as expected, report less cognitive impairment and have lower biomarker levels. Second, among SCD subjects the most frequent impaired domain was memory, and the most frequent risk factors were air pollution (due to our specific setting in Lombardy, where the levels of PM10 and PM2.5 significantly exceed the 2021 WHO Air Quality Guidelines [25, 26]), sleep issues, smoking, physical inactivity, and hypertension. Third, up to a third of SCD individuals had plasma Lumipulse p-Tau217 above cut-off, indicating an increased probability of underlying amyloid pathology. For group comparisons, participants were analytically classified as having plasma Lumipulse p-Tau217 values above (A+) or below (A−) the prespecified cut-off of 0.15 pg/mL, derived from an independent analysis in a SCD sample [14], and should be regarded as an operational definition within the present study. It should not be interpreted as confirmed amyloid status. We observed significant differences between A+ and A− individuals with SCD. A+ subjects were on average older, had lower qMCI scores, worse hearing, and higher Lancet RR compared to A− subjects. A+ individuals also exhibited higher biomarkers of amyloid-β, neuroinflammation, and neurodegeneration, as well as more atrophy on MRI visual scales. Finally, disclosure was feasible and well-accepted, although it resulted in a slightly higher psychological impact in A+ subjects, well below the threshold for PTSD.

Our results on FCD align with the literature on the topic. In particular, it is well recognized that individuals with FCD are younger, typically of working age – indeed, in our cohort they are in their sixth decade – and report more language impairment [27]. They also experience affective symptoms [28]. Contrary to what is described in previous works [29], in our cohort the performance on MoCA was normal, and closer to that of WW and SCD rather than to that of MCI. This might be due to the different setting, as previous works were done in memory clinics, which might result in a population of more severe FCD compared to our BHS. FCD and WW groups represent individuals who “self-filtered” into a service marketed for brain health. Their presence is a finding in itself, demonstrating that 10–12% of people seeking dementia prevention actually have functional or psychological drivers. Given our exploratory findings in these groups, it could be argued that the BHS might serve a differential diagnostic role, identifying subjects who may benefit more from reassurance than from amyloid-targeted prevention.

Our results confirm the clinical heterogeneity of SCD. Up to a third of subjects may harbor preclinical AD, and these have early signatures of ongoing pathophysiological processes, including amyloid deposition, neurodegeneration and neuroinflammation. These findings support the relevance of SCD as a transitional stage between normal cognition and MCI in the AD continuum [4]. Notably, almost half of A− SCD subjects considered their memory worse than peers, compared to only a fifth of A + SCD subjects. This paradoxical finding suggests the existence of distinct metacognitive profiles, characterized by heightened self-awareness in A− individuals and an early erosion of insight among A+ subjects, consistent with emerging evidence on anosognosia in preclinical stages [31, 32].

The cluster analysis identified two distinct clusters that differed in age, biomarker levels, and cognitive performance. Cluster 1 included younger participants with lower levels of neurodegeneration markers and higher cognitive scores, whereas Cluster 2 consisted of older participants with elevated plasma GFAP, NfL, and p-Tau217, as well as lower cognitive performance. These findings align with current models of preclinical and prodromal neurodegenerative processes, where biomarker accumulation is accompanied by cognitive decline. GFAP, NfL, and p-Tau217 were the primary contributors to cluster differentiation, supporting their potential as combinatorial markers of neurodegeneration. The robustness of clustering after excluding p-Tau217 indicates that the observed structure reflects a multi-marker biological profile rather than reliance on a single biomarker. Overall, the clusters represent biologically plausible subgroups, with Cluster 2 reflecting a higher-risk profile characterized by elevated neurodegeneration markers and reduced cognitive function, which may have implications for early detection, risk stratification, and targeted interventions. Moreover, the greater stability of the high-risk Cluster 2 suggests that individuals with concomitant elevations in plasma biomarkers, modifiable risk burden, and older age constitute a well-defined subgroup. Conversely, the lower stability of the low-risk Cluster 1 likely reflects the greater biological and clinical heterogeneity of individuals without this high-risk profile. Although amyloid status offers a binary classification, the clustering approach incorporates age, cognitive measures, and multiple biomarkers to generate a multidimensional risk profile, which may facilitate more precise stratification of individuals with SCD. Cluster 2 may thus represent a higher-risk profile characterized by elevated neurodegeneration markers and reduced cognitive function, which can facilitate a more precise stratification for targeted interventions beyond just amyloid-lowering therapies. Consistent with this interpretation, recent findings from the large SCD SCIENCe cohort demonstrated that baseline plasma GFAP and NfL, as well as longitudinal changes in these biomarkers, were associated with subsequent cognitive decline independently of amyloid status, supporting the concept that dementia risk in SCD is better captured through multidimensional biomarker profiles rather than amyloid status alone [33].

Disclosure of biomarkers is a delicate and sensitive topic [5]. The protocol for disclosure has been harmonized within the ICOPAD BHS network, and follows expert opinions and specific recommendations [20, 34]. Previous works show that disclosure of amyloid-PET results in SCD subjects had a non-trivial psychological impact, which was however below the threshold for clinical concern [35]. In our BHS, we use the same scale, the IES-R. Even though a difference emerged between A+ and A− post-disclosure, this did not survive FDR correction, and the median score in A+ (11.50 (7.25, 21.25)) was well below the threshold for post-traumatic stress disorder (IES-*R* = 33). The marginal unadjusted difference in IES-R total scores between A + and A– was no longer evident after adjustment for age and APOE 4 carriage. Thus, the observed psychological response cannot be specifically attributed to disclosure of the p-Tau217 result. Age and APOE 4 carriage were associated with higher IES-R scores in an exploratory model, although the limited sample size and simultaneous disclosure of multiple risk-related results preclude causal interpretation. Moreover, participants showed a high level of satisfaction, and felt that the disclosure was informative and useful. Finally, no differences were observed between A+ and A − in affective measures following disclosure. Overall, these findings suggest that biomarker disclosure in a real-world BHS setting is generally well tolerated and perceived as useful in the short term. No significant increase in anxiety or depression was observed, although A+ individuals exhibited a modest rise in event-related distress. This response appears to represent a typical emotional reaction to risk information rather than clinically significant psychological harm. This extends what already present in the literature on AD biomarker disclosure in preclinical phases [6, 36–40]; however, while almost all previous studies disclosed amyloid-PET or CSF results in research settings, we disclosed blood-based biomarker results and dementia risk in a clinical setting, which requires specific ethical and communication considerations [20, 34]. Following the IWG conservative approach for SCD, we disclosed the plasma p-Tau217 result and its associated uncertainty as part of the participant’s risk profile. Above-cut-off results were communicated as a risk factor for future decline, and not as a definitive diagnosis [5]. Moreover, testing was conducted within a prospective observational cohort study, corresponding to the research context in which the AA Workgroup does not recommend against biomarker investigation in cognitively unimpaired individuals [4]. Our finding that biomarker disclosure was well-tolerated (with median IES-R scores in A+ subjects far below clinical thresholds) provides real-world evidence for the safety of transparent risk communication in a preventative setting. This safety is likely attributable to the disclosure protocol’s emphasis on the uncertainty and modifiability of risk, following the literature on the ethics of risk communication for cognitively unimpaired individuals [41]. Risk perception decreased after disclosure among A–, whereas it remained unchanged among those receiving an above-cut-off, indeterminate result. Although the between-group difference was only marginal after FDR correction, this pattern may indicate that disclosure primarily reassured A–, without substantially changing perceived risk among those receiving an above cut-off result. Longer follow-up is needed to determine whether risk communication produces sustained lifestyle changes. However, by framing A+ not as an inevitable disease but as a “risk multiplier” (calculated via the Brookmeyer method and the Lancet RR composite), we shift the focus towards secondary prevention. While the biological profile of A + SCD individuals aligns with findings from established research cohorts [14], its identification within a functioning BHS demonstrates the feasibility of translating biomarker-based risk stratification into preventive care. Unlike curated research registries, our BHS captures a heterogeneous real-world population, where clinically meaningful risk profiles are more challenging to identify but highly relevant. Our main contributions include preliminary evidence of reduced insight in the preclinical stage, and a multimarker clustering approach extending beyond binary amyloid classification to identify a stable phenotype of “multidimensional brain vulnerability”, integrating neuroinflammation (GFAP), axonal injury (NfL), cognitive performance, and modifiable risk factors. Finally, we showed the feasibility of biomarker disclosure and risk communication within a clinical preventive framework, extending previous evidence from research settings.

Our work has some limitations. First, formal neuropsychological assessment was not performed systematically in all participants classified as SCD, and test-level data from assessments conducted in the memory clinic were not available for the present analyses. Although formal neuropsychological assessment confirmed the classification of participants included in the MCI group and was also performed when the SCD–MCI distinction was clinically uncertain, subtle objective cognitive impairment may have remained undetected in some participants classified as SCD. Possible misclassification of the primary analytical sample should therefore be considered when interpreting its clinical and biomarker characteristics. Second, the sample size was low, especially for the comparisons between A + and A − SCD subjects. Third, the cluster analysis was done on a modest sample size with a cross-sectional design, which prevents conclusions about progression over time that could clarify the interpretation of the clusters. Fourth, the multiplicative Lancet RR should be regarded a proxy for cumulative risk burden rather than a definitive epidemiological forecast. Fifth, because plasma p-Tau217, APOE genotype, and the individualized dementia-risk estimate were disclosed during the same visit, their respective psychological effects cannot be disentangled. The observed IES-R responses should therefore be interpreted as responses to the combined disclosure encounter rather than specifically to the plasma p-Tau217 result. The multivariable analysis with age and APOE status was exploratory. Finally, biomarker disclosure was available only for half of the subjects with biomarkers available (*n* = 84), which limits the validity of the conclusions drawn on the topic. Given the limited sample size and short follow-up period, these results should be interpreted with caution. Further longitudinal studies are required to more comprehensively assess the medium- and long-term psychological effects of disclosure and to calibrate risk estimates against actual conversion rates to MCI or dementia. To overcome these limitations, a large multicenter prospective observational cohort study, lead by our BHS, is undergoing, pooling data from several BHS to characterize the SCD population.

However, our work also has strengths. It presents a cohort of subjects seen in a BHS, which is a new setting, different from that of the memory clinics. Indeed, the current cohort is among the largest cohorts of BHS in Europe. Moreover, SCD subjects in our BHS are extensively characterized in terms of risk factors, plasma biomarkers and neuroimaging, covering a comprehensive set of aspects related to their clinical and biological picture.

In conclusion, our findings showed a high prevalence of modifiable risk factors in SCD, supporting the central role of BHS in prevention. A+ individuals demonstrated biological and structural hallmarks of early neurodegeneration. Biomarker disclosure was feasible and well-accepted in the short term, with minimal psychological effects, with no evidence of clinically significant psychological harm in this small sample. These findings support incorporating plasma biomarkers and structured disclosure protocols in BHS, complementing multimodal prevention strategies to delay dementia onset.

## Supplementary Information

Below is the link to the electronic supplementary material.

Supplementary Material 1

## Data Availability

The data of this manuscript are available upon request to the corresponding author.
