# Supplementary material for: Dementia risk factors and biomarkers in subjective cognitive decline: real world evidence from the monza brain health service
Source: Neurol Sci. 2026 Sep 29;47(10):824. doi: 10.1007/s10072-026-09426-1 (PMC13624042; doi:10.1007/s10072-026-09426-1)
Supplement: Supplementary file 1 [file 10072_2026_9426_MOESM1_ESM.docx]

**Supplementary material**

**Dementia risk factors and biomarkers in Subjective Cognitive Decline: real world evidence from a Brain Health Service**

Table of Contents:

[Supplementary Methods	2](#_Toc238666249)

[Operational classification of SCD and MCI	2](#_Toc238666250)

[Disclosure protocol	2](#_Toc238666251)

[Risk reduction protocol	2](#_Toc238666252)

[References	4](#_Toc238666253)

[Supplementary Figures	5](#_Toc238666254)

[Fig. S1 Diagnosis distribution in BHS in the whole BHS cohort (n=186, panel A) and in the subset with available plasma biomarkers (n=115, panel B)	5](#_Toc238666255)

[Fig. S2 Silhouette method for optimal clusters	6](#_Toc238666256)

[Fig. S3 Radar chart of cluster profiles for k-means cluster analyses	7](#_Toc238666257)

[Supplementary Tables	8](#_Toc238666258)

[Table S1 Additional characteristics of the whole cohort participants	8](#_Toc238666259)

[Table S2 Additional differences between A+ and A- SCD subjects	11](#_Toc238666260)

[Table S3 Variable differences between clusters	14](#_Toc238666261)

[Table S4 Variable differences between clusters in A- SCD subjects	15](#_Toc238666262)

[Table S5 Additional biomarker disclosure-related differences between A+ and A- SCD subjects	16](#_Toc238666263)

This supplementary material has been provided by the authors to give readers additional information about their work.

# Supplementary Methods

## *Operational classification of SCD and MCI*

Diagnostic categories were assigned by the evaluating neurologist on the basis of the referral diagnosis, detailed clinical history, subjective and informant-reported cognitive changes when available, preservation of independence in everyday activities, and performance on brief cognitive screening measures, including the MoCA and qMCI. Participants reporting cognitive decline, with preserved functional independence and no clinically evident objective impairment on this integrated assessment, were operationally classified as SCD. Participants showing clinically evident cognitive impairment on screening, while retaining substantial independence in everyday activities, were referred to the memory clinic for formal neuropsychological assessment. Formal neuropsychological testing confirmed the MCI classification in all participants ultimately included in the MCI group. No single MoCA or qMCI threshold was used as an automatic classification rule, but subjects were referred to formal neuropsychological testing when they had MoCA < 26 and qMCI < 49.5. When the distinction between SCD and MCI remained uncertain, participants were also referred for formal neuropsychological testing, and the final clinical classification was based on the results of that assessment. However, test-level neuropsychological data were not available in the analytical dataset. Because formal neuropsychological testing was not performed systematically in all participants classified as SCD, the absence of subtle objective cognitive impairment could not be verified in the entire SCD group.

## *Disclosure protocol*

The disclosure protocol generally follows what described in the relevant BHS publications [1,2] and the ICOPAD network workshop on the topic held in 2025 (https://www.icopad.ch/en/).

Briefly, the disclosure begins with an assessment of the subject’s perception and understanding of their dementia risk, allowing the clinician to identify and address any knowledge gaps using simple, non-technical language. Risk estimates are then communicated using absolute numbers and a clearly defined timeframe (e.g., "Among 100 people with the same risk profile as yours, approximately five are expected to develop dementia within the next 10 years."). When appropriate and requested by the participant, the clinician also provides a qualitative interpretation of the estimated risk (e.g., explaining that a 5% risk over 20 years can be considered low and therefore reassuring). Risk estimates may also be contextualized by comparison with the lifetime risk of more familiar conditions, such as breast or lung cancer.

Risk is visually presented using a dedicated shinyapp that displays the relative contribution of non-modifiable and modifiable risk factors with curves of different colors. This tool helps explain how individual risk factors contribute to the overall estimated risk. Each modifiable risk factor is discussed in detail, while emphasizing that the estimated percentages represent probabilistic, point estimates and are therefore subject to uncertainty. Participants are also informed that additional risk factors, including some not considered in the Lancet Commission model [3], such as sleep impairment or cognitive reserve, may increase or lower dementia risk but cannot currently be quantified.

To facilitate understanding and avoid framing bias, risk is communicated using both positive (e.g., "Five out of 100 people with your risk profile are expected to develop dementia.") and complementary negative framing (e.g., "This also means that 95 out of 100 people with your risk profile are not expected to develop dementia.").

Among the measured biomarkers, only plasma p-Tau217 and APOE genotype are disclosed because they have sufficient clinical interpretability in the SCD population [4]. In particular, a cut-off of 0.15 pg/mL for plasma Lumipulse p-Tau217 has a 98% NPV and a 64% PPV to detect amyloid positivity, as shown in an independent cohort [4]. As such, plasma Lumipulse p-Tau217 below the cut-off makes AD pathology unlikely, while a result above the cut-off results in an “indeterminate” interpretation, which is fully disclosed to the subject. Other biomarkers, including NfL and GFAP, are measured for research purposes but are not disclosed, as their clinical significance in individuals with SCD remains insufficiently established.

If participants express interest, especially after an indeterminate result of Lumipulse plasma p-Tau217, additional diagnostic investigations, such as lumbar puncture for CSF AD biomarker assessment, may be offered. However, this is uncommon in routine BHS clinical practice and is reserved for selected cases following careful clinical evaluation.

One week after disclosure, participants complete follow-up online questionnaires, including the Impact of Event Scale–Revised (IES-R) and the HADS, which is compared with pre-disclosure HADS. Psychological support is available upon request or whenever the clinician considers it appropriate. In our cohort, no participant required formal psychological support, nor was it judged necessary by the clinical team. One participant requested an additional disclosure visit to further discuss the implications of the biomarker results and reported feeling reassured after the consultation.

## *Risk reduction protocol*

Risk reduction mostly follows what recommended in the relevant BHS paper [2], adapted to the specific landscape of our institution.

Briefly, rather than prescribing a standardized multidomain intervention as in the FINGER model [5], preventive strategies are individualized according to each participant's modifiable risk factors. For example, participants reporting sleep disturbances are referred to a specialized sleep medicine center for further assessment, including polysomnography when clinically indicated, and appropriate sleep counselling. Other lifestyle interventions, such as dietary modifications aimed at weight reduction and increased physical activity, are recommended but are not delivered through a structured referral pathway. Participants are therefore free to choose the healthcare professionals who will support these interventions, although these services are generally not covered by the public healthcare system and may require out-of-pocket payment. If other treatable medical conditions are identified, such as hearing or vision impairment, diabetes, hypertension, or other vascular risk factors, participants are referred to the appropriate specialist for further evaluation and management.

Participants who express interest may also be offered the opportunity to participate in ongoing research studies investigating multidomain lifestyle interventions, cognitive enhancement, cognitive stimulation and socialization [6], and tailored physical activity. The results of these intervention studies are currently under evaluation and will be reported separately.

## *References*

1.	Visser LNC, Minguillon C, Sánchez-Benavides G, Abramowicz M, Altomare D, Fauria K, et al. Dementia risk communication. A user manual for Brain Health Services—part 3 of 6. Alzheimers Res Ther. 2021;13(1):170. doi: 10.1186/S13195-021-00840-5

2.	Frisoni GB, Altomare D, Ribaldi F, Villain N, Brayne C, Mukadam N, et al. Dementia prevention in memory clinics: recommendations from the European task force for brain health services. The Lancet Regional Health - Europe. 2023;26:100576. doi: 10.1016/j.lanepe.2022.100576

3.	Livingston G, Huntley J, Liu KY, Costafreda SG, Selbæk G, Alladi S, et al. Dementia prevention, intervention, and care: 2024 report of the Lancet standing Commission. The Lancet. 2024;404(10452):572–628. doi: 10.1016/S0140-6736(24)01296-0

4.	Contador J, Pozzi FE, Sánchez-Benavides G, Fernández-Arcos A, Lantero-Rodríguez J, Montoliu-Gaya L, et al. Detecting amyloid–β pathology in subjective cognitive decline using plasma and CSF biomarkers. EBioMedicine. 2026;129:106331. doi: 10.1016/j.ebiom.2026.106331

5.	Ngandu T, Lehtisalo J, Solomon A, Levälahti E, Ahtiluoto S, Antikainen R, et al. A 2 year multidomain intervention of diet, exercise, cognitive training, and vascular risk monitoring versus control to prevent cognitive decline in at-risk elderly people (FINGER): a randomised controlled trial. The Lancet. 2015;385(9984):2255–63. doi: 10.1016/S0140-6736(15)60461-5

6.	Pozzi FE, Spanio A, Gallo F, Isgrò G, Remoli G, Magi A, et al. Cognitive and social intervention with Go and chess in early and subjective cognitive decline: The COGniChESs study results, with an updated meta-analysis. J Alzheimers Dis. 2026;109(2):747–56. doi: 10.1177/13872877251401481

# Supplementary Figures

## Fig. S1 Diagnosis distribution in BHS in the whole BHS cohort (n=186, panel A) and in the subset with available plasma biomarkers (n=115, panel B)

Abbreviations: FCD - Functional cognitive disorder; MCI – Mild cognitive impairment; SCD – Subjective cognitive decline; WW – Worried well

## Fig. S2 Silhouette method for optimal clusters

## Fig. S3 Radar chart of cluster profiles for k-means cluster analyses

# Supplementary Tables

## Table S1 Additional characteristics of the whole cohort participants

|  | **Whole cohort** | | **WW** | | **FCD** | | **SCD** | | **MCI** | |  |  |  |
| --- | --- | --- | --- | --- | --- | --- | --- | --- | --- | --- | --- | --- | --- |
| **Variable** | **n** | **Distribution** | **n** | **Distribution** | **N** | **Distribution** | **n** | **Distribution** | **n** | **Distribution** | ***P*** | ***P_adj_*** | ***P_FDR_*** |
| *Clinical variables* | | | | | | | | | | | | | |
| Familial history of dementia | 184 | 114 (61.96%) | 17 | 12 (70.59%) | 18 | 12 (66.67%) | 130 | 80 (61.54%) | 19 | 10 (52.63%) | 0.701 |  | 0.773 |
| Peers comparison | 185 | better: 20 (10.81%);  same: 104 (56.22%);  worse: 61 (32.97%) | 17 | better: 6 (35.29%);  same: 11 (64.71%);  worse: 0 (0.00%) | 18 | better: 1 (5.56%);  same: 1 (5.56%);  worse: 16 (88.89%) | 131 | better: 12 (9.16%);  same: 80 (61.07%);  worse: 39 (29.77%) | 19 | better: 1 (5.26%);  same: 12 (63.16%);  worse: 6 (31.58%) | <0.001 |  | <0.001 |
| Languages spoken | 129 | 1.00 (1.00, 2.00) | 17 | 1.00 (1.00, 2.00) | 12 | 1.00 (1.00, 2.25) | 88 | 1.00 (1.00, 2.00) | 12 | 1.00 (1.00, 1.00) | 0.338 |  | 0.501 |
| *Risk and resilience factors* | | | | | | | | | | | | | |
| Low education | 186 | 9 (4.84%) | 17 | 0 (0.00%) | 18 | 1 (5.56%) | 132 | 6 (4.55%) | 19 | 2 (10.53%) | 0.523 |  | 0.679 |
| Hear loss | 186 | 40 (21.51%) | 17 | 1 (5.88%) | 18 | 3 (16.67%) | 132 | 31 (23.48%) | 19 | 5 (26.32%) | 0.351 |  | 0.509 |
| Whisper test | 128 | 5.00 (3.50, 5.50) | 15 | 5.50 (4.50, 6.00) | 11 | 5.50 (5.00, 6.00) | 90 | 4.75 (3.62, 5.50) | 12 | 3.50 (2.38, 5.12) | 0.042 |  | 0.095 |
| Whisper < 4 | 128 | 43 (33.59%) | 15 | 3 (20.00%) | 11 | 2 (18.18%) | 90 | 31 (34.44%) | 12 | 7 (58.33%) | 0.125 |  | 0.236 |
| High total cholesterol | 162 | 83 (51.23%) | 15 | 9 (60.00%) | 16 | 11 (68.75%) | 115 | 55 (47.83%) | 16 | 8 (50.00%) | 0.396 |  | 0.548 |
| Total cholesterol | 162 | 202.00 (176.00, 220.00) | 15 | 202.00 (194.00, 229.50) | 16 | 218.50 (199.00, 235.50) | 115 | 200.00 (169.00, 220.00) | 16 | 201.00 (172.75, 216.25) | 0.098 |  | 0.199 |
| High LDL | 118 | 46 (38.98%) | 13 | 4 (30.77%) | 11 | 7 (63.64%) | 84 | 30 (35.71%) | 10 | 5 (50.00%) | 0.254 |  | 0.398 |
| LDL | 118 | 124.00 (97.50, 140.75) | 13 | 123.00 (116.00, 138.00) | 11 | 134.00 (113.00, 152.50) | 84 | 122.00 (87.50, 137.25) | 10 | 129.50 (94.00, 141.75) | 0.381 |  | 0.535 |
| HDL | 118 | 61.50 (53.00, 75.75) | 13 | 56.00 (53.00, 61.00) | 11 | 76.00 (60.00, 85.50) | 84 | 62.00 (52.75, 75.00) | 10 | 54.90 (48.75, 65.75) | 0.159 |  | 0.281 |
| Tryglicerids | 116 | 89.50 (66.75, 123.00) | 13 | 98.00 (59.00, 115.00) | 11 | 103.00 (89.50, 119.50) | 82 | 88.50 (66.25, 123.00) | 10 | 88.50 (61.00, 111.00) | 0.491 |  | 0.653 |
| Mood impairment | 186 | 92 (49.46%) | 17 | 7 (41.18%) | 18 | 15 (83.33%) | 132 | 59 (44.70%) | 19 | 11 (57.89%) | 0.015 |  | 0.041 |
| Past depression | 131 | 14 (10.69%) | 15 | 2 (13.33%) | 12 | 0 (0.00%) | 92 | 11 (11.96%) | 12 | 1 (8.33%) | 0.621 |  | 0.737 |
| HADS - anxiety | 111 | 4.00 (3.00, 6.00) | 11 | 3.00 (2.50, 5.00) | 11 | 7.00 (5.50, 9.50) | 76 | 4.00 (3.00, 6.00) | 13 | 3.00 (2.00, 6.00) | 0.018 |  | 0.049 |
| HADS - depression | 111 | 5.00 (3.00, 6.00) | 11 | 3.00 (3.00, 5.00) | 11 | 7.00 (4.50, 8.00) | 76 | 5.00 (3.75, 6.00) | 13 | 5.00 (5.00, 5.00) | 0.046 |  | 0.100 |
| HADS - total | 111 | 9.00 (7.00, 12.00) | 11 | 8.00 (5.00, 9.50) | 11 | 12.00 (11.00, 16.00) | 76 | 9.00 (7.00, 12.00) | 13 | 8.00 (7.00, 11.00) | 0.007 |  | 0.024 |
| Traumatic brain injury | 186 | 20 (10.75%) | 17 | 1 (5.88%) | 18 | 1 (5.56%) | 132 | 16 (12.12%) | 19 | 2 (10.53%) | 0.756 |  | 0.815 |
| Physical inactivity | 185 | 94 (50.81%) | 17 | 3 (17.65%) | 18 | 12 (66.67%) | 131 | 65 (49.62%) | 19 | 14 (73.68%) | 0.004 |  | 0.016 |
| Diabetes | 186 | 11 (5.91%) | 17 | 0 (0.00%) | 18 | 1 (5.56%) | 132 | 8 (6.06%) | 19 | 2 (10.53%) | 0.614 |  | 0.737 |
| HbA1c | 100 | 5.45 (5.20, 5.70) | 11 | 5.40 (5.20, 5.40) | 10 | 5.50 (5.32, 5.80) | 69 | 5.40 (5.20, 5.70) | 10 | 5.60 (5.50, 5.75) | 0.356 |  | 0.509 |
| Ischemic cardiopathy | 132 | 4 (3.03%) | 15 | 0 (0.00%) | 12 | 0 (0.00%) | 93 | 4 (4.30%) | 12 | 0 (0.00%) | 0.630 |  | 0.737 |
| Previous stroke | 118 | 3 (2.54%) | 14 | 0 (0.00%) | 11 | 0 (0.00%) | 81 | 3 (3.70%) | 12 | 0 (0.00%) | 0.704 |  | 0.773 |
| Current smoker | 181 | 90 (49.72%) | 17 | 7 (41.18%) | 18 | 6 (33.33%) | 127 | 65 (51.18%) | 19 | 12 (63.16%) | 0.271 |  | 0.417 |
| Pack-years | 181 | 0.00 (0.00, 13.00) | 17 | 0.00 (0.00, 5.00) | 18 | 0.00 (0.00, 1.95) | 127 | 0.30 (0.00, 15.00) | 19 | 4.00 (0.00, 17.50) | 0.202 |  | 0.335 |
| Hypertension | 186 | 77 (41.40%) | 17 | 4 (23.53%) | 18 | 5 (27.78%) | 132 | 60 (45.45%) | 19 | 8 (42.11%) | 0.211 |  | 0.344 |
| Obesity | 182 | 23 (12.64%) | 17 | 3 (17.65%) | 18 | 3 (16.67%) | 129 | 15 (11.63%) | 18 | 2 (11.11%) | 0.848 |  | 0.868 |
| Overweight | 182 | 72 (39.56%) | 17 | 7 (41.18%) | 18 | 9 (50.00%) | 129 | 49 (37.98%) | 18 | 7 (38.89%) | 0.807 |  | 0.837 |
| BMI | 182 | 24.24 (22.33, 27.41) | 17 | 24.44 (22.79, 27.70) | 18 | 25.58 (22.93, 28.91) | 129 | 24.22 (22.04, 27.10) | 18 | 24.09 (22.65, 27.21) | 0.571 |  | 0.707 |
| Excessive alcohol | 127 | 4 (3.15%) | 14 | 0 (0.00%) | 11 | 0 (0.00%) | 88 | 4 (4.55%) | 14 | 0 (0.00%) | 0.608 |  | 0.737 |
| Weekly alcohol units | 127 | 1.00 (0.00, 6.00) | 14 | 3.25 (1.00, 5.50) | 11 | 0.00 (0.00, 3.50) | 88 | 1.00 (0.00, 6.00) | 14 | 3.00 (1.00, 7.00) | 0.197 |  | 0.334 |
| LSNS | 111 | 14.00 (11.00, 18.00) | 11 | 16.00 (11.50, 21.00) | 11 | 16.00 (11.50, 17.50) | 76 | 13.00 (11.00, 18.00) | 13 | 15.00 (8.00, 20.00) | 0.649 |  | 0.749 |
| Past isolation | 111 | 40 (36.04%) | 11 | 5 (45.45%) | 11 | 9 (81.82%) | 76 | 23 (30.26%) | 13 | 3 (23.08%) | 0.006 |  | 0.021 |
| Air pollution | 186 | 186 (100.00%) | 17 | 17 (100.00%) | 18 | 18 (100.00%) | 132 | 132 (100.00%) | 19 | 19 (100.00%) | 1.000 |  | 1.000 |
| Vision loss | 186 | 10 (5.38%) | 17 | 0 (0.00%) | 18 | 1 (5.56%) | 132 | 8 (6.06%) | 19 | 1 (5.26%) | 0.780 |  | 0.830 |
| Hyposmia | 117 | 24 (20.51%) | 14 | 0 (0.00%) | 11 | 6 (54.55%) | 80 | 17 (21.25%) | 12 | 1 (8.33%) | 0.006 |  | 0.021 |
| Atrial fibrillation | 185 | 7 (3.78%) | 17 | 2 (11.76%) | 18 | 0 (0.00%) | 131 | 3 (2.29%) | 19 | 2 (10.53%) | 0.077 |  | 0.163 |
| Hypothyroidism | 149 | 24 (16.11%) | 17 | 0 (0.00%) | 13 | 2 (15.38%) | 102 | 20 (19.61%) | 17 | 2 (11.76%) | 0.219 |  | 0.349 |
| eGFR (CKD.EPI) | 91 | 82.00 (72.00, 89.50) | 11 | 81.00 (77.00, 89.50) | 11 | 87.00 (81.00, 95.00) | 59 | 80.00 (72.00, 89.00) | 10 | 82.50 (74.00, 88.00) | 0.547 |  | 0.698 |
| CAIDE | 71 | 5.00 (4.00, 6.00) | 9 | 6.00 (5.00, 7.00) | 15 | 6.00 (4.00, 6.00) | 47 | 5.00 (4.00, 6.00) | 0 | NA | 0.495 |  | 0.653 |
| BDSI | 102 | 12.50 (6.25, 17.00) | 8 | 2.50 (0.75, 4.75) | 2 | 20.00 (19.50, 20.50) | 78 | 11.50 (7.00, 17.00) | 14 | 19.50 (14.00, 23.50) | <0.001 |  | <0.001 |
| LIBRA | 114 | 1.50 (0.58, 2.58) | 14 | 1.50 (0.40, 1.67) | 7 | 3.60 (3.35, 3.85) | 81 | 1.50 (0.40, 2.50) | 12 | 2.20 (1.50, 6.45) | 0.010 |  | 0.031 |
| Risk factor count | 186 | 3.00 (2.00, 5.00) | 17 | 3.00 (2.00, 3.00) | 18 | 4.00 (3.00, 4.75) | 132 | 3.00 (2.00, 5.00) | 19 | 4.00 (3.00, 5.50) | 0.025 |  | 0.058 |
| Sleep issues | 186 | 95 (51.08%) | 17 | 9 (52.94%) | 18 | 14 (77.78%) | 132 | 67 (50.76%) | 19 | 5 (26.32%) | 0.020 |  | 0.051 |
| OSAS | 186 | none: 74 (39.78%);  snoring: 82 (44.09%);  apneas or OSAS: 30 (16.13%) | 17 | none: 7 (41.18%);  snoring: 7 (41.18%);  apneas or OSAS: 3 (17.65%) | 18 | none: 7 (38.89%);  snoring: 9 (50.00%);  apneas or OSAS: 2 (11.11%) | 132 | none: 52 (39.39%);  snoring: 58 (43.94%);  apneas or OSAS: 22 (16.67%); | 19 | none: 8 (42.11%);  snoring: 8 (42.11%);  apneas or OSAS: 3 (15.79%) | 0.997 |  | 0.997 |
| RBD | 185 | no RBD: 171 (92.43%);  suspected: 13 (7.03%); probable/confirmed: 1 (0.54%) | 17 | no RBD: 16 (94.12%);  suspected: 1 (5.88%);  probable/confirmed: 0 (0.00%) | 18 | no RBD: 16 (88.89%);  suspected: 2 (11.11%);  probable/confirmed: 0 (0.00%) | 131 | no RBD: 120 (91.60%);  suspected: 10 (7.63%);  probable/confirmed: 1 (0.76%) | 19 | no RBD: 19 (100.00%);  suspected: 0 (0.00%)  probable/confirmed: 0 (0.00%); | 0.876 |  | 0.887 |
| PSQI | 129 | 8.00 (5.00, 11.00) | 14 | 6.00 (4.25, 9.50) | 12 | 10.00 (6.75, 12.25) | 87 | 8.00 (5.00, 11.00) | 16 | 6.50 (2.75, 11.00) | 0.130 |  | 0.240 |
| ESS | 129 | 4.00 (2.00, 7.00) | 14 | 3.00 (2.00, 5.00) | 12 | 6.00 (3.75, 11.50) | 87 | 4.00 (2.00, 7.50) | 16 | 6.00 (2.75, 6.25) | 0.193 |  | 0.333 |
| PSS | 129 | 14.00 (9.00, 19.00) | 14 | 10.50 (5.50, 13.75) | 12 | 22.00 (19.75, 24.50) | 87 | 14.00 (9.00, 19.00) | 16 | 13.00 (10.50, 18.25) | 0.001 |  | 0.007 |
| MEDILITE | 129 | 8.00 (7.00, 10.00) | 14 | 8.00 (7.00, 9.00) | 12 | 9.00 (8.00, 9.00) | 87 | 8.00 (7.00, 10.00) | 16 | 8.50 (8.00, 10.25) | 0.702 |  | 0.773 |
| BRS | 111 | 3.50 (2.83, 4.00) | 11 | 3.83 (2.67, 4.00) | 11 | 2.50 (2.33, 3.92) | 76 | 3.50 (2.83, 4.00) | 13 | 3.67 (3.00, 4.00) | 0.468 |  | 0.637 |
| Hobby | 185 | 51 (27.57%) | 17 | 2 (11.76%) | 18 | 2 (11.11%) | 131 | 42 (32.06%) | 19 | 5 (26.32%) | 0.116 |  | 0.228 |
| GOHAI | 111 | 51.00 (42.00, 58.00) | 11 | 57.00 (47.00, 59.00) | 11 | 55.00 (46.50, 58.00) | 76 | 51.00 (40.00, 56.25) | 13 | 46.00 (44.00, 59.00) | 0.316 |  | 0.476 |
| CRIq - school | 110 | 114.00 (108.00, 123.00) | 11 | 120.00 (107.50, 122.50) | 11 | 118.00 (107.00, 121.50) | 75 | 114.00 (108.50, 123.00) | 13 | 114.00 (102.00, 117.00) | 0.564 |  | 0.707 |
| CRIq - work | 111 | 105.00 (93.50, 116.50) | 11 | 123.00 (114.50, 132.50) | 11 | 100.00 (89.00, 102.50) | 76 | 107.00 (92.50, 117.00) | 13 | 99.00 (90.00, 105.00) | <0.001 |  | 0.005 |
| CRIq - leisure | 110 | 135.50 (120.00, 151.50) | 11 | 137.00 (126.00, 151.00) | 11 | 133.00 (127.00, 136.50) | 75 | 137.00 (120.50, 153.00) | 13 | 141.00 (114.00, 150.00) | 0.798 |  | 0.837 |
| CRIq - total | 110 | 125.00 (114.00, 134.00) | 11 | 134.00 (129.00, 144.50) | 11 | 119.00 (116.50, 121.50) | 75 | 126.00 (115.00, 135.00) | 13 | 113.00 (107.00, 131.00) | 0.023 |  | 0.055 |
| *Cognitive variables* | | | | | | | | | | | | | |
| ECOG | 129 | 1.50 (1.25, 1.92) | 14 | 1.17 (1.08, 1.46) | 12 | 1.75 (1.33, 2.44) | 87 | 1.42 (1.25, 1.75) | 16 | 1.92 (1.63, 2.10) | <0.001 | 0.002 | 0.005 |
| Altered CDT | 180 | 35 (19.44%) | 17 | 1 (5.88%) | 18 | 3 (16.67%) | 126 | 20 (15.87%) | 19 | 11 (57.89%) | <0.001 |  | 0.001 |
| Hand praxis | 180 | 4.00 (3.00, 4.00) | 17 | 4.00 (3.00, 4.00) | 17 | 4.00 (3.00, 4.00) | 129 | 4.00 (3.00, 4.00) | 17 | 3.00 (3.00, 3.00) | <0.001 | <0.001 | 0.003 |
| Recent facts | 178 | 2.00 (2.00, 2.00) | 17 | 2.00 (2.00, 2.00) | 18 | 2.00 (2.00, 2.00) | 125 | 2.00 (2.00, 2.00) | 18 | 1.50 (1.00, 2.00) | <0.001 | <0.001 | <0.001 |
| *Genetics* | | | | | | | | | | | | | |
| APOE | 115 | 23: 5 (4.35%);  24: 1 (0.87%);  33: 72 (62.61%);  34: 34 (29.57%);  44: 3 (2.61%) | 12 | 23: 1 (8.33%);  24: 0 (0.00%);  33: 8 (66.67%);  34: 1 (8.33%);  44: 2 (16.67%) | 6 | 23: 0 (0.00%);  24: 0 (0.00%);  33: 4 (66.67%);  34: 2 (33.33%);  44: 0 (0.00%) | 84 | 23: 4 (4.76%);  24: 0 (0.00%);  33: 53 (63.10%);  34: 27 (32.14%);  44: 0 (0.00%) | 13 | 23: 0 (0.00%);  24: 1 (7.69%);  33: 7 (53.85%);  34: 4 (30.77%);  44: 1 (7.69%) | 0.020 |  | 0.051 |
| APOE4 carrier | 115 | 38 (33.04%) | 12 | 3 (25.00%) | 6 | 2 (33.33%) | 84 | 27 (32.14%) | 13 | 6 (46.15%) | 0.707 |  | 0.773 |

Characteristics of the whole cohort. Data are expressed as median with interquartile range or percentage (%). Differences among diagnostic groups were tested with the Kruskal-Wallis rank sum test or a Pearson’s *χ²* test, depending on the type of variable. Cognitive variables were compared adjusting for age, sex, and education (*P_adj_*).

*P-*values were adjusted for multiple comparisons with FDR (*P_FDR_)*.

Due to limited sample sizes, comparisons involving FCD, MCI and WW groups are descriptive and hypothesis-generating only. Statistical values for these rows should be interpreted with caution.

Abbreviations: ESS, Epworth Sleepiness Scale; HADS, Hospital Anxiety and Depression Scale; LSNS, Lubben Social Network Scale; PSQI, Pittsburgh Sleep Quality Index; PSS, Perceived Stress Scale

## Table S2 Additional differences between A+ and A- SCD subjects

|  | **Whole SCD cohort** | | **A-** | | **A+** | |  |  |  |  |
| --- | --- | --- | --- | --- | --- | --- | --- | --- | --- | --- |
| **Variable** | **n** | **Distribution** | **n** | **Distribution** | **n** | **Distribution** | ***P*** | ***P_adj_*** | ***P_FDR_*** | ***r*** |
| *Clinical variables* | | | | | | | | | | |
| Complaints duration (years) | 84 | 2.50 (2.00, 4.00) | 57 | 2.00 (2.00, 4.00) | 27 | 4.00 (2.00, 5.00) | 0.173 | 0.379 | 0.458 | 0.18 |
| Peers comparison | 84 | better: 8 (9.52%);  same: 48 (57.14%);  worse: 28 (33.33%) | 57 | better: 7 (12.28%);  same: 27 (47.37%);  worse: 23 (40.35%) | 27 | better: 1 (3.70%);  same: 21 (77.78%);  worse: 5 (18.52%) | 0.030 |  | 0.153 |  |
| Familial history of dementia | 84 | 51 (60.71%) | 57 | 35 (61.40%) | 27 | 16 (59.26%) | 1.000 |  | 1.000 |  |
| Languages spoken | 71 | 2.00 (1.00, 2.50) | 49 | 2.00 (1.00, 3.00) | 22 | 1.50 (1.00, 2.00) | 0.692 | 0.835 | 0.962 | -0.06 |
| *Complaints* | | | | | | | | | | |
| Memory | 84 | 83 (98.81%) | 57 | 56 (98.25%) | 27 | 27 (100.00%) | 1.000 |  | 1.000 |  |
| Attention | 84 | 46 (54.76%) | 57 | 28 (49.12%) | 27 | 18 (66.67%) | 0.203 |  | 0.498 |  |
| Orientation | 84 | 21 (25.00%) | 57 | 13 (22.81%) | 27 | 8 (29.63%) | 0.686 |  | 0.962 |  |
| Language | 84 | 60 (71.43%) | 57 | 41 (71.93%) | 27 | 19 (70.37%) | 1.000 |  | 1.000 |  |
| Autonomy | 84 | 8 (9.52%) | 57 | 5 (8.77%) | 27 | 3 (11.11%) | 1.000 |  | 1.000 |  |
| *Risk factors* | | | | | | | | | | |
| Low education | 84 | 1 (1.19%) | 57 | 1 (1.75%) | 27 | 0 (0.00%) | 1.000 |  | 1.000 |  |
| Hypoacusia | 84 | 21 (25.00%) | 57 | 13 (22.81%) | 27 | 8 (29.63%) | 0.686 |  | 0.962 |  |
| Whisper < 4 | 81 | 28 (34.57%) | 55 | 14 (25.45%) | 26 | 14 (53.85%) | 0.024 |  | 0.136 |  |
| High total cholesterol | 79 | 41 (51.90%) | 54 | 31 (57.41%) | 25 | 10 (40.00%) | 0.231 |  | 0.539 |  |
| Total cholesterol | 79 | 203.00 (168.00, 222.50) | 54 | 205.50 (176.25, 232.25) | 25 | 190.00 (162.00, 214.00) | 0.113 | 0.113 | 0.383 | -0.22 |
| High LDL | 76 | 28 (36.84%) | 52 | 21 (40.38%) | 24 | 7 (29.17%) | 0.492 |  | 0.845 |  |
| LDL | 76 | 125.50 (90.25, 140.50) | 52 | 125.50 (104.75, 138.50) | 24 | 124.00 (77.50, 142.75) | 0.444 | 0.381 | 0.818 | -0.11 |
| HDL | 76 | 62.50 (53.75, 75.25) | 52 | 65.50 (57.75, 79.25) | 24 | 58.50 (46.00, 68.50) | 0.055 | 0.063 | 0.239 | -0.28 |
| Triglycerids | 74 | 88.00 (66.00, 122.75) | 50 | 80.50 (64.25, 121.75) | 24 | 102.50 (83.50, 133.00) | 0.072 | 0.659 | 0.287 | 0.26 |
| Depression | 84 | 4 (4.76%) | 57 | 2 (3.51%) | 27 | 2 (7.41%) | 0.814 |  | 1.000 |  |
| Mood impairment | 84 | 35 (41.67%) | 57 | 23 (40.35%) | 27 | 12 (44.44%) | 0.906 |  | 1.000 |  |
| Past depression | 81 | 10 (12.35%) | 55 | 5 (9.09%) | 26 | 5 (19.23%) | 0.351 |  | 0.742 |  |
| HADS - anxiety | 72 | 4.00 (3.00, 6.00) | 48 | 4.00 (3.00, 6.25) | 24 | 4.50 (2.75, 6.00) | 0.966 | 0.958 | 1.000 | 0.01 |
| HADS - depression | 72 | 5.00 (3.00, 6.00) | 48 | 5.00 (4.00, 6.00) | 24 | 4.00 (3.00, 6.00) | 0.158 | 0.791 | 0.444 | -0.20 |
| HADS - total | 72 | 9.00 (7.00, 12.00) | 48 | 9.50 (7.00, 12.00) | 24 | 8.00 (6.50, 13.00) | 0.388 | 0.872 | 0.784 | -0.13 |
| Traumatic brain injury | 84 | 10 (11.90%) | 57 | 7 (12.28%) | 27 | 3 (11.11%) | 1.000 |  | 1.000 |  |
| Physical inactivity | 84 | 34 (40.48%) | 57 | 28 (49.12%) | 27 | 6 (22.22%) | 0.035 |  | 0.168 |  |
| Diabetes | 84 | 4 (4.76%) | 57 | 3 (5.26%) | 27 | 1 (3.70%) | 1.000 |  | 1.000 |  |
| HbA1c | 64 | 5.45 (5.20, 5.70) | 42 | 5.50 (5.23, 5.70) | 22 | 5.40 (5.23, 5.70) | 0.744 | 0.693 | 1.000 | 0.00 |
| Ischemic cardiopathy | 82 | 3 (3.66%) | 56 | 2 (3.57%) | 26 | 1 (3.85%) | 1.000 |  | 1.000 |  |
| Previous stroke | 78 | 3 (3.85%) | 54 | 2 (3.70%) | 24 | 1 (4.17%) | 1.000 |  | 1.000 |  |
| Current smoker | 84 | 47 (55.95%) | 57 | 31 (54.39%) | 27 | 16 (59.26%) | 0.853 |  | 1.000 |  |
| Pack-years | 84 | 2.00 (0.00, 15.00) | 57 | 1.25 (0.00, 15.00) | 27 | 5.00 (0.00, 19.88) | 0.400 | 0.822 | 0.791 | 0.11 |
| Hypertension | 84 | 31 (36.90%) | 57 | 18 (31.58%) | 27 | 13 (48.15%) | 0.220 |  | 0.526 |  |
| Obesity | 84 | 6 (7.14%) | 57 | 5 (8.77%) | 27 | 1 (3.70%) | 0.697 |  | 0.962 |  |
| Overweight | 84 | 31 (36.90%) | 57 | 19 (33.33%) | 27 | 12 (44.44%) | 0.457 |  | 0.818 |  |
| BMI | 84 | 24.12 (21.23, 26.39) | 57 | 23.57 (20.83, 25.62) | 27 | 24.61 (21.31, 27.11) | 0.301 | 0.229 | 0.685 | 0.14 |
| Excessive alcohol | 45 | 2 (4.44%) | 27 | 1 (3.70%) | 18 | 1 (5.56%) | 1.000 |  | 1.000 |  |
| Weekly alcohol units | 45 | 2.00 (1.00, 7.00) | 27 | 2.00 (1.00, 6.00) | 18 | 3.00 (1.00, 12.25) | 0.468 | 0.768 | 0.818 | 0.13 |
| Social isolation | 84 | 22 (26.19%) | 57 | 13 (22.81%) | 27 | 9 (33.33%) | 0.448 |  | 0.818 |  |
| LSNS | 72 | 13.00 (11.00, 18.00) | 48 | 13.50 (11.00, 18.00) | 24 | 13.00 (11.25, 17.25) | 0.981 | 0.487 | 1.000 | 0.00 |
| Past isolation | 72 | 22 (30.56%) | 48 | 15 (31.25%) | 24 | 7 (29.17%) | 1.000 |  | 1.000 |  |
| Air pollution | 84 | 1: 84 (100.00%) | 57 | 1: 57 (100.00%) | 27 | 1: 27 (100.00%) | 1.000 |  | 1.000 |  |
| Vision loss | 84 | 4 (4.76%) | 57 | 3 (5.26%) | 27 | 1 (3.70%) | 1.000 |  | 1.000 |  |
| Hyposmia | 77 | 17 (22.08%) | 53 | 12 (22.64%) | 24 | 5 (20.83%) | 1.000 |  | 1.000 |  |
| Atrial fibrillation | 84 | 2 (2.38%) | 57 | 1 (1.75%) | 27 | 1 (3.70%) | 1.000 |  | 1.000 |  |
| Hypothyroidism | 83 | 18 (21.69%) | 56 | 10 (17.86%) | 27 | 8 (29.63%) | 0.350 |  | 0.742 |  |
| eGFR (CKD.EPI) | 55 | 81.00 (72.00, 89.00) | 37 | 84.00 (73.00, 90.00) | 18 | 74.50 (62.50, 82.75) | 0.030 | 0.148 | 0.153 | -0.36 |
| CAIDE | 34 | 5.00 (5.00, 6.00) | 29 | 5.00 (5.00, 6.00) | 5 | 6.00 (5.00, 7.00) | 0.114 | 0.079 | 0.383 | 0.43 |
| BDSI | 51 | 10.00 (6.50, 15.50) | 29 | 8.00 (4.00, 16.00) | 22 | 12.50 (9.00, 14.00) | 0.085 | 0.530 | 0.321 | 0.29 |
| LIBRA | 75 | 1.50 (0.40, 2.50) | 49 | 1.50 (0.40, 2.50) | 26 | 1.95 (1.18, 3.03) | 0.139 | 0.311 | 0.409 | 0.21 |
| Risk factors count | 84 | 3.00 (2.00, 5.00) | 57 | 3.00 (2.00, 5.00) | 27 | 3.00 (3.00, 5.00) | 0.586 | 0.290 | 0.920 | 0.07 |
| Sleep issues | 84 | 42 (50.00%) | 57 | 32 (56.14%) | 27 | 10 (37.04%) | 0.161 |  | 0.444 |  |
| OSAS | 84 | none: 30 (35.71%);  snoring: 36 (42.86%)  apneas or OSAS: 18 (21.43%); | 57 | none: 18 (31.58%);  snoring: 23 (40.35%)  apneas or OSAS: 16 (28.07%); | 27 | none: 12 (44.44%);  snoring: 13 (48.15%)  apneas or OSAS: 2 (7.41%); | 0.093 |  | 0.337 |  |
| RBD | 84 | no RBD: 77 (91.67%);  suspected: 6 (7.14%)  probable/confirmed: 1 (1.19%); | 57 | no RBD: 52 (91.23%);  suspected: 4 (7.02%)  probable/confirmed: 1 (1.75%); | 27 | no RBD: 25 (92.59%);  suspected: 2 (7.41%)  probable/confirmed: 0 (0.00%); | 0.786 |  | 1.000 |  |
| PSQI | 76 | 8.00 (5.00, 11.25) | 49 | 8.00 (5.00, 11.00) | 27 | 8.00 (4.00, 12.00) | 0.794 | 0.348 | 1.000 | -0.04 |
| ESS | 76 | 4.00 (2.00, 7.25) | 49 | 4.00 (2.00, 7.00) | 27 | 4.00 (3.00, 7.00) | 0.592 | 0.159 | 0.920 | 0.07 |
| PSS | 76 | 14.00 (9.00, 19.00) | 49 | 13.00 (9.00, 19.00) | 27 | 16.00 (11.00, 18.50) | 0.514 | 0.390 | 0.852 | 0.09 |
| MEDILITE | 76 | 9.00 (7.00, 10.00) | 49 | 9.00 (8.00, 10.00) | 27 | 8.00 (7.00, 10.00) | 0.596 | 0.254 | 0.920 | -0.07 |
| BRS | 72 | 3.67 (3.00, 4.00) | 48 | 3.67 (3.00, 4.00) | 24 | 3.50 (2.96, 4.00) | 0.334 | 0.077 | 0.742 | -0.14 |
| Hobby | 84 | 5 (5.95%) | 57 | 2 (3.51%) | 27 | 3 (11.11%) | 0.378 |  | 0.782 |  |
| GOHAI | 72 | 51.00 (41.00, 57.00) | 48 | 52.50 (42.75, 56.25) | 24 | 50.00 (39.75, 58.25) | 0.649 | 0.797 | 0.962 | -0.07 |
| CRIq - school | 71 | 114.00 (109.50, 123.50) | 48 | 114.00 (108.00, 124.00) | 23 | 114.00 (112.50, 123.00) | 0.610 |  | 0.925 | 0.08 |
| CRIq - work | 72 | 108.00 (90.75, 117.25) | 48 | 108.00 (92.50, 117.00) | 24 | 109.50 (89.75, 118.75) | 0.938 |  | 1.000 | 0.01 |
| CRIq - leisure | 71 | 137.00 (121.00, 153.00) | 48 | 141.50 (125.25, 155.75) | 23 | 129.00 (116.50, 148.50) | 0.064 |  | 0.266 | -0.27 |
| CRIq - total | 71 | 127.00 (116.00, 135.00) | 48 | 129.00 (119.00, 135.25) | 23 | 123.00 (113.00, 133.50) | 0.176 |  | 0.458 | -0.20 |
| *Cognitive variables* | | | | | | | | | |  |
| ECOG | 76 | 1.42 (1.25, 1.83) | 49 | 1.33 (1.25, 1.75) | 27 | 1.50 (1.29, 1.83) | 0.427 | 0.950 | 0.818 | 0.11 |
| Altered CDT | 83 | 11 (13.25%) | 56 | 6 (10.71%) | 27 | 5 (18.52%) | 0.524 |  | 0.852 |  |
| Hand praxis | 83 | 4.00 (3.00, 4.00) | 56 | 4.00 (3.00, 4.00) | 27 | 4.00 (3.00, 4.00) | 0.782 | 0.892 | 1.000 | -0.03 |
| Recent facts | 83 | 2.00 (2.00, 2.00) | 56 | 2.00 (2.00, 2.00) | 27 | 2.00 (2.00, 2.00) | 0.123 | 0.111 | 0.385 | -0.11 |
| *MRI – visual scales* | | | | | | | | | | |
| MTA [L] | 46 | 0.00 (0.00, 1.00) | 28 | 0.00 (0.00, 1.00) | 18 | 1.00 (0.25, 2.00) | 0.001 | 0.109 | 0.012 | 0.51 |
| MTA [R] | 46 | 0.00 (0.00, 1.00) | 28 | 0.00 (0.00, 1.00) | 18 | 1.00 (0.25, 1.00) | 0.008 | 0.411 | 0.055 | 0.42 |
| PCA [L] | 46 | 1.00 (0.00, 1.00) | 28 | 0.00 (0.00, 1.00) | 18 | 1.00 (1.00, 2.00) | <0.001 | 0.017 | 0.003 | 0.60 |
| PCA [R] | 46 | 1.00 (0.00, 1.00) | 28 | 0.00 (0.00, 1.00) | 18 | 1.00 (1.00, 2.00) | <0.001 | 0.008 | <0.001 | 0.65 |

Characteristics of the SCD cohort. Data are expressed as median with interquartile range or percentage (%). A+ was defined as plasma p-Tau217 ≥ 0.15 pg/mL, measured by Lumipulse (Fujirebio). Differences between A+ and A- groups were tested with the Wilcoxon rank sum test or a Pearson’s *χ²* test, depending on the type of variable. Continuous clinical variables, risk factors (except for CRIq, which is already intrinsically corrected), and MRI visual scales *P-*values were compared adjusting for age (*P_adj_*). Cognitive variables were compared adjusting for age, sex, and education (*P_adj_*). *P-*values were adjusted for multiple comparisons with FDR. Effect sizes have been computed with rank-biserial correlations (r).

Abbreviations: ESS, Epworth Sleepiness Scale; GCA, Global Cortical Atrophy; HADS, Hospital Anxiety and Depression Scale; LSNS, Lubben Social Network Scale; MTA, Medial Temporal Atrophy; PCA, Parietal Cortical Atrophy; PSQI, Pittsburgh Sleep Quality Index; PSS, Perceived Stress Scale

## Table S3 Variable differences between clusters

| **Variable** | **Cluster 1 (n=51)** | **Cluster 2 (n=30)** | ***P*** |
| --- | --- | --- | --- |
| Age | 62.00 (58.00, 67.50) | 72.00 (70.00, 76.00) | <0.001 |
| GFAP | 33.60 (27.40, 39.65) | 64.75 (50.30, 93.27) | <0.001 |
| Lancet RR | 2.97 (1.79, 4.05) | 5.61 (3.32, 7.38) | <0.001 |
| NfL | 14.12 (10.57, 16.66) | 20.34 (16.08, 26.56) | <0.001 |
| pTau217 | 0.10 (0.07, 0.11) | 0.17 (0.11, 0.36) | <0.001 |
| qMCI | 68.50 (61.50, 74.75) | 57.50 (48.12, 62.88) | <0.001 |

Differences between clusters were tested with the Wilcoxon rank sum test

## Table S4 Variable differences between clusters in A- SCD subjects

| **Variable** | **Cluster 1 (n=45)** | **Cluster 2 (n=10)** | ***P*** | ***P_FDR_*** |
| --- | --- | --- | --- | --- |
| Age | 62.00 (59.00-68.00) | 70.00 (69.25-71.00) | <0.001 | <0.001 |
| qMCI | 69.00 (62.00-75.00) | 58.00 (51.38-63.38) | 0.004 | 0.053 |
| Lancet RR | 2.91 (1.65-3.99) | 6.88 (3.19-11.47) | 0.012 | 0.060 |
| p-Tau217 | 0.09 (0.07-0.11) | 0.09 (0.09-0.11) | 0.377 | 0.838 |
| GFAP | 32.90 (27.00-39.90) | 51.20 (37.70-54.88) | 0.008 | 0.053 |
| NfL | 14.55 (9.88-17.54) | 17.13 (14.55-22.60) | 0.020 | 0.080 |
| Sex (Female) | 31 (68.9%) | 7 (70.0%) | 1.000 | 1.000 |
| Low education | 0 (0.0%) | 1 (10.0%) | 0.182 | 0.607 |
| Hypertension | 10 (22.2%) | 7 (70.0%) | 0.006 | 0.053 |
| Diabetes | 2 (4.4%) | 1 (10.0%) | 0.459 | 0.915 |
| Obesity | 3 (6.7%) | 2 (20.0%) | 0.220 | 0.629 |
| Smoker | 22 (48.9%) | 7 (70.0%) | 0.303 | 0.758 |
| Physical inactivity | 21 (46.7%) | 6 (60.0%) | 0.503 | 0.915 |
| Depression | 1 (2.2%) | 0 (0.0%) | 1.000 | 1.000 |
| Hear loss | 11 (24.4%) | 3 (30.0%) | 0.703 | 1.000 |
| Social isolation | 11 (28.2%) | 3 (33.3%) | 1.000 | 1.000 |
| Excessive alcohol | 1 (5.0%) | 0 (0.0%) | 1.000 | 1.000 |
| Air pollution | 45 (100.0%) | 10 (100.0%) | 1.000 | 1.000 |
| High LDL | 18 (41.9%) | 3 (37.5%) | 1.000 | 1.000 |
| Traumatic brain injury | 5 (11.1%) | 1 (10.0%) | 1.000 | 1.000 |
| Vision loss | 3 (6.7%) | 0 (0.0%) | 1.000 | 1.000 |

Differences between clusters were tested with the Wilcoxon rank sum test or a Pearson’s *χ²* test, depending on the type of variable. *P-*values were adjusted for multiple comparisons with FDR.

## Table S5 Additional biomarker disclosure-related differences between A+ and A- SCD subjects

|  | **Whole SCD cohort** | | **A-** | | **A+** | |  |  |
| --- | --- | --- | --- | --- | --- | --- | --- | --- |
| **Variable** | **n** | **Distribution** | **n** | **Distribution** | **n** | **Distribution** | ***P*** | ***P_FDR_*** |
| Informant [present] | 42 | 30 (71.43%) | 32 | 24 (75.00%) | 10 | 6 (60.00%) | 0.606 | 0.738 |
| Need for psychological support | 42 | 1 (2.38%) | 32 | 1 (3.12%) | 10 | 0 (0.00%) | 1.000 | 1.000 |
| Research participation motivation | 42 | 0.00 (0.00, 1.00) | 32 | 0.00 (0.00, 1.00) | 10 | 0.50 (0.00, 1.00) | 0.628 | 0.738 |
| Usefulness | 42 | 1: 42 (100.00%) | 32 | 1: 32 (100.00%) | 10 | 1: 10 (100.00%) | 1.000 | 1.000 |
| Report perception | 42 | clear: 36 (85.71%);  difficult: 5 (11.90%);  uninformative: 1 (2.38%) | 32 | clear: 27 (84.38%);  difficult: 4 (12.50%);  uninformative: 1 (3.12%) | 10 | clear: 9 (90.00%);  difficult: 1 (10.00%);  uninformative: 0 (0.00%) | 0.827 | 0.930 |
| HADS - anxiety, pre-disclosure | 38 | 4.00 (3.00, 7.00) | 30 | 4.50 (3.00, 7.75) | 8 | 3.50 (2.00, 5.00) | 0.086 | 0.212 |
| HADS - anxiety, post-disclosure | 42 | 3.00 (2.00, 6.00) | 32 | 3.00 (2.00, 6.00) | 10 | 3.00 (1.25, 4.50) | 0.420 | 0.567 |
| HADS - depression, pre-disclosure | 38 | 5.00 (3.00, 6.00) | 30 | 5.00 (4.00, 6.00) | 8 | 3.00 (2.00, 5.00) | 0.031 | 0.094 |
| HADS - depression, post-disclosure | 42 | 4.00 (3.00, 5.00) | 32 | 4.00 (3.00, 5.00) | 10 | 4.00 (2.25, 4.00) | 0.330 | 0.496 |
| HADS - total, pre-disclosure | 38 | 9.00 (7.00, 12.00) | 30 | 10.00 (7.25, 13.00) | 8 | 7.00 (4.50, 8.25) | 0.015 | 0.056 |
| HADS - total, post-disclosure | 42 | 7.00 (5.00, 10.00) | 32 | 7.00 (5.75, 10.25) | 10 | 6.00 (5.00, 8.75) | 0.358 | 0.508 |

Differences in disclosure variables between A+ and A- SCD. Data are expressed as median with interquartile range or percentage (%). A+ was defined as plasma p-Tau217 ≥ 0.15 pg/mL, measured by Lumipulse (Fujirebio). Differences between A+ and A- groups were tested with the Wilcoxon rank sum test or a Pearson’s χ² test, depending on the type of variable. *P*-values were adjusted for multiple comparisons with FDR.

Abbreviations: HADS, Hospital Anxiety and Depression Scale; IES-R. Impact of event scale – revised. ·
